# Comparative valorization of olive pomace *via* ZnCl_2_ and H_3_PO_4_ activation: toward high-performance adsorbents for phenol removal

**DOI:** 10.1039/d6ra04727k

**Published:** 2026-09-01

**Authors:** Badreddine Djemmali, Nadjib Dahdouh, Haroun Hafsa, Annabel Dias Fernandes, Venkataraman Sivasankar, Mohammed Kebir, Imane Kezrane, Amina Ouarghi, Toufik Chaabane

**Affiliations:** a Reaction Engineering Laboratory, University of Science and Technology Houari Boumediene B.P.32, El-Alia Bab-Ezzouar Algeria nadjibdahdouh@outlook.com; b Scientific and Technical Center of Research in Physical and Chemical Analysis CRAPC BP384, Bou-Ismail 42004 Tipaza Algeria; c Department of Chemistry, Fiber Materials and Environmental Technologies (FibEnTech-UBI), Universidade da Beira Interior, R. Marquês D’Ávila e Bolama 6201-001 Covilhã Portugal; d Graduate School of Integrated Science and Technology, Nagasaki University 1 – 14 Bunkyo-machi Nagasaki 852 8521 Japan

## Abstract

This study focuses on the valorization of olive pomace from an olive oil extraction industry in Kabylia (Algeria) for the sustainable production of high performance activated carbons. Olive pomace was chemically activated using ZnCl_2_ and H_3_PO_4_, followed by carbonization at 500 °C, yielding OP-AC(ZC) and OP-AC(PA) respectively. This work presents a novel comparative approach by evaluating two widely used activating agents under identical preparation conditions, allowing a direct assessment of their influence on the physicochemical properties and adsorption performance of the resulting materials. Comprehensive characterization (SEM-EDS, BET, XRD, FTIR) revealed a significant enhancement of textural properties after activation. BET surface areas increased from 48 m^2^ g^−1^ (biochar) to 430.9 m^2^ g^−1^ for OP-AC(ZC) and 1012.6 m^2^ g^−1^ for OP-AC(PA). The point of zero charge (pHpzc) was determined at 6.34 and 3.85, respectively. XRD analysis confirmed the predominantly amorphous nature of the carbons with slight structural differences induced by the activation agents, while FTIR and SEM-EDS results evidenced the presence of oxygen- and phosphorus-/zinc-containing functional groups contributing to surface reactivity. A parametric study on phenol adsorption demonstrated that the natural solution pH offered the highest removal efficiency for both adsorbents. The optimum adsorption performance was achieved at the natural solution pH (≈6.5), using adsorbent doses of 30 mg for OP-AC(ZC) and 20 mg for OP-AC(PA), highlighting the favorable effect of near-neutral conditions and appropriate adsorbent dosage on phenol removal. The adsorption performance was further influenced by adsorbent dose and initial phenol concentration, highlighting the role of surface availability and mass transfer limitations. Increasing the initial phenol concentration enhanced adsorption capacity while reducing removal efficiency. Adsorption modelling using Langmuir, Freundlich, Temkin and Dubinin–Radushkevich isotherms showed that the Langmuir model best described the adsorption process. Maximum monolayer capacities reached 143.54 mg g^−1^ for OP-AC(ZC) and 269.03 mg g^−1^ for OP-AC(PA). Comparative analysis with literature data indicates that the prepared materials exhibit competitive to superior adsorption capacities, particularly for the H_3_PO_4_-activated carbon. Overall, the results confirm that olive pomace is an efficient low-cost precursor for producing activated carbon with excellent performance toward phenol removal.

## Abstract 2

This study focuses on the valorization of olive pomace from an olive oil extraction industry in Kabylia (Algeria) for the sustainable production of high performance activated carbons.

## Graphical abstract

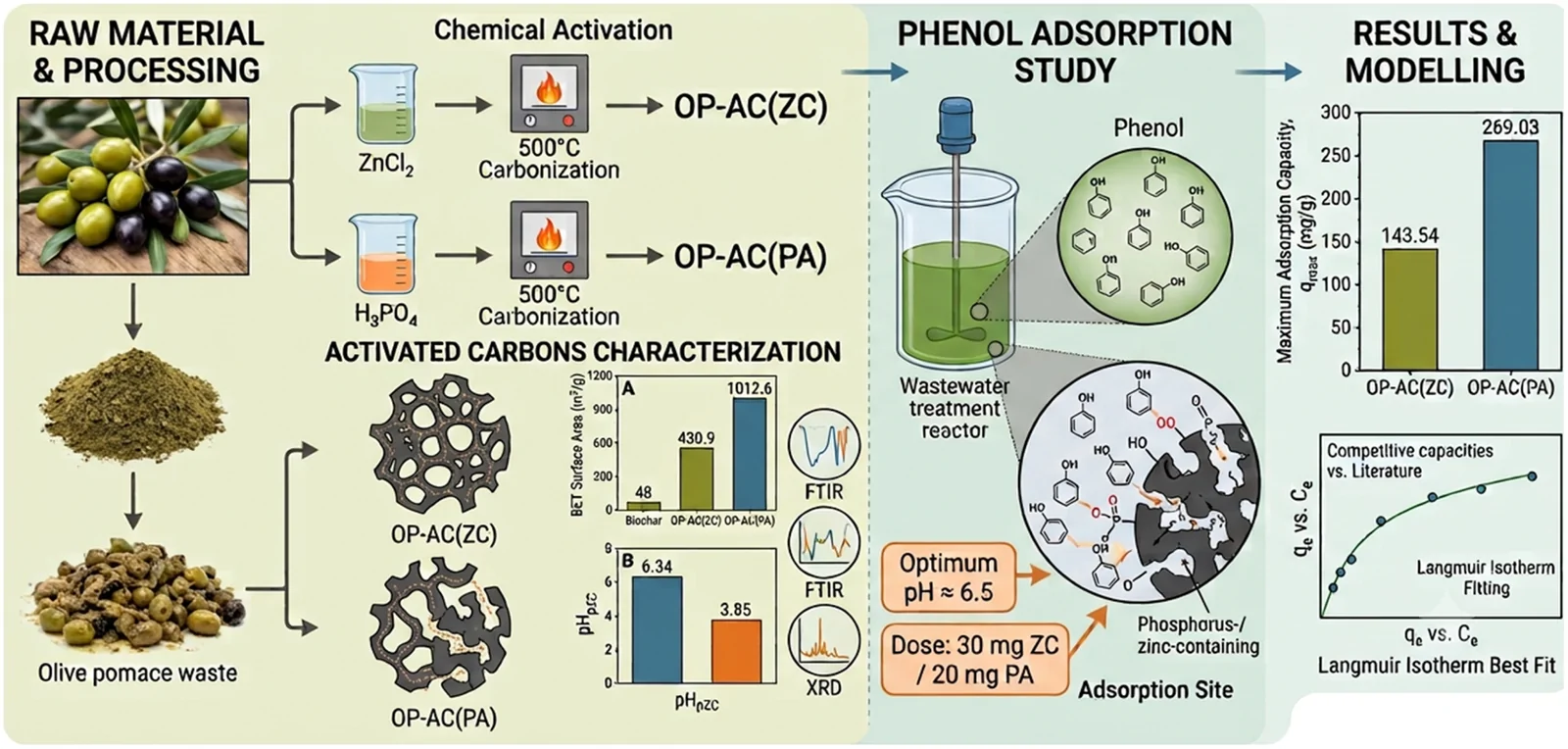

## 1 Introduction

Phenolic compounds, particularly phenol (C_6_H_5_OH), represent one of the most hazardous groups of organic pollutants due to their high solubility in water, marked toxicity, and limited biodegradability. They are widely generated from various industrial processes such as petroleum refining, coal conversion, petrochemical production, resin synthesis, and pulp and paper manufacturing, which collectively discharge significant quantities of phenol into aquatic environments.^1–3^ Because of their aromatic structure, phenolic compounds exhibit strong resistance to natural degradation, allowing them to persist for extended periods in water bodies.^1,4^ Numerous ecotoxicological investigations have shown that phenol and cresols cause severe adverse effects on aquatic organisms, including algae, crustaceans, and fish, mainly through membrane damage, metabolic disruption, and oxidative stress.^1,4^ In humans, phenol exposure is associated with acute and chronic health effects, including hepatotoxicity, nephrotoxicity, neurotoxicity, and potential mutagenic or carcinogenic impacts.^2,5^ For these reasons, phenol is listed among priority pollutants by many environmental protection agencies, and stringent limits are imposed on industrial wastewater discharges.^6^ Although several treatment technologies such as biological processes, advanced oxidation processes, membrane filtration, solvent extraction, and adsorption have been developed, many remain costly, generate secondary contaminants, or show reduced performance at high phenol concentrations.^2,7^ Adsorption is widely recognized as one of the most effective and versatile techniques for wastewater treatment due to its high removal efficiency, simple operation, low energy consumption, and adaptability to a broad range of contaminants. Compared with conventional treatment technologies, adsorption offers significant advantages, including the absence of harmful by-products, ease of process design and operation, and the possibility of using sustainable, low-cost adsorbents derived from agricultural and industrial wastes. These features have promoted its extensive application for the removal of phenolic compounds and other refractory organic pollutants from aqueous media. Recent advances in adsorbent development and process optimization have further strengthened the role of adsorption as a promising and environmentally friendly technology for large-scale water purification applications.^8,9^ Consequently, the development of efficient, low-cost, and environmentally sustainable methods for phenol removal from wastewater remains a critical research priority. The olive oil industry generates large quantities of olive pomace, a by-product composed of residual pulp, skin fragments, and stones, which poses significant environmental challenges if not managed properly. Recent work emphasizes the urgency of valorizing this waste stream due to its high organic load and phenolic content, which can lead to soil and water contamination.^10^ Activation and transformation of olive pomace into high-performance adsorbents align with principles of circular economy and sustainability. Recent studies have demonstrated that olive pomace can be converted into activated carbon (AC) or biochar with remarkable adsorption properties. For instance, Mohamed Abdoul-Latif *et al.* (2023) produced a biochar from olive pomace that achieved an adsorption capacity of ∼66.7 mg g^−1^ for total phenols in olive mill effluent and showed favorable thermodynamic behavior, being spontaneous and exothermic.^10^ Optimization studies have also been conducted. Ait Merzeg *et al.* (2023) used response surface methodology to optimize phenol adsorption onto pomace-derived activated carbon and reported removal efficiencies up to 91% under optimal conditions.^11^ In addition, continuous systems have been explored: Lissaneddine *et al.* (2021) investigated a packed-bed reactor using pomace-derived AC beads for dynamic phenol recovery from olive mill wastewater, showing promising stability and regeneration potential.^12^ Beyond conventional activation, chemical modification and material engineering have been applied: de Oliveira Rocha *et al.* (2024) synthesized a variety of adsorbents from olive pomace and olive stone (biochars, chemically-modified AC) and demonstrated high adsorption capacities for phenolic acids typical of olive mill effluent.^13^ Thermodynamic and kinetic modeling has also advanced: Melo *et al.* (2024) provided a mass-transfer and equilibrium framework for phenol adsorption on agro-waste derived AC, confirming the spontaneous and entropy-driven nature of the process.^14^ Finally, hybrid materials such as composites are emerging: Abu-Dalo *et al.* (2023) developed a composite of granular activated carbon and a copper-based metal–organic framework (MOF), which effectively removed phenolic compounds from olive mill wastewater under mild conditions.^15^ These developments strongly support the potential of olive pomace as a sustainable and high-performance precursor for designing novel adsorbents tailored for phenol removal and other phenolic pollutants in aqueous media. Recent studies continue to confirm the strong potential of olive pomace-derived activated carbons. A recent study (2026) reported that activated carbons derived from olive pomace exhibit very high adsorption capacities under semi-industrial conditions, highlighting their suitability for large-scale applications.^16^ In addition, recent works (2025) demonstrated their effectiveness for the removal of both organic and inorganic contaminants, with high performance and good stability.^17^ Very recent studies (2024) have further shown that optimizing chemical activation parameters significantly improves the porosity and adsorption efficiency of these materials, making them competitive with commercial adsorbents.^18^ Chemical activation of olive pomace using agents like H_3_PO_4_ or ZnCl_2_ has proven effective for producing activated carbons with highly developed porosity. For example, María Lorena *et al.* (2025) developed activated carbons from desilicated rice husks using carbonization and chemical activation with phosphoric acid and calcium carbonate. The materials exhibited a highly developed porous structure, and the best sample achieved an iodine number of 1094.8 mg g^−1^, confirming their strong potential for groundwater purification and the valorization of agricultural waste as sustainable adsorbents.^19^ Other works report that ZnCl_2_ activation can yield more microporous structures, beneficial for small molecule adsorption such as phenols.^20^ Activation conditions (*e.g.*, impregnation agent concentration, temperature, time) strongly influence the textural and chemical properties of the resulting carbon, which in turn affects its adsorption capacity. These studies highlight that chemical activation is a key step in converting olive pomace into efficient, low-cost adsorbents for water treatment. Among the various chemical activating agents reported for lignocellulosic biomass, ZnCl_2_ and H_3_PO_4_ are the two most extensively employed owing to their high activation efficiency and their distinct effects on pore development and surface chemistry.^21,22^ ZnCl_2_ mainly acts as a dehydrating agent, promoting the degradation of cellulose and hemicellulose while enhancing aromatization during carbonization, which generally results in a predominantly microporous carbon structure.^21^ In contrast, H_3_PO_4_ promotes bond cleavage, inhibits tar formation, and facilitates the development of both micropores and mesopores while introducing oxygen- and phosphorus-containing functional groups on the carbon surface.^21,22^ These complementary activation mechanisms often lead to markedly different physicochemical properties and adsorption behaviors. Therefore, a direct comparison of ZnCl_2_ and H_3_PO_4_ under identical preparation conditions is scientifically relevant to elucidate how the activation chemistry governs pore architecture and ultimately controls the adsorption performance of olive pomace-derived activated carbons. In the present study, two activated carbons were synthesized from olive pomace using ZnCl_2_ and H_3_PO_4_ as activating agents in order to evaluate and compare their performance in phenol adsorption from aqueous media. A detailed parametric study was carried out to investigate the influence of pH, contact time, initial phenol concentration, adsorbent dosage, and temperature on the adsorption process. The equilibrium behavior of phenol on both activated carbons was analyzed using the most widely applied adsorption isotherm models, namely Langmuir, Freundlich, Temkin, and Dubinin–Radushkevich (D–R), allowing a deeper understanding of the adsorption mechanism. Extensive structural and surface characterizations including XRD, SEM–EDS, BET and FTIR were performed before and after adsorption to identify the physicochemical changes induced by phenol uptake. These analyses helped clarify the dominant interactions responsible for phenol removal by each activated carbon. Finally, the obtained results were compared with recent literature, demonstrating the promising potential of olive-pomace-derived activated carbons for phenol elimination. In addition to evaluating the adsorption performance of olive pomace-derived activated carbons, this work provides new insights into the influence of the chemical activation route on the physicochemical characteristics and adsorption behavior of the resulting materials. Unlike previous studies, which generally investigated a single activating agent or employed different preparation conditions, the present study systematically compares ZnCl_2_ and H_3_PO_4_ under identical synthesis parameters, allowing the specific effect of the activating agent to be isolated. This comparative approach establishes a direct relationship between the activation chemistry, pore development, surface functionalization, and phenol adsorption performance. The findings therefore contribute to a better understanding of how the choice of activating agent governs the structural and adsorption properties of biomass-derived activated carbons, providing useful guidance for the rational design of sustainable adsorbents for wastewater treatment.

## 2 Materials and methods

### 2.1. Reagents and materials

All chemicals used in this study were of analytical grade and applied without further purification.Sodium hydroxide (NaOH, crystalline, ≥99.5%, Sigma-Aldrich, USA), potassium hydroxide (KOH, pellets, ≥85%, Merck, Germany), hydrochloric acid (HCl, 37%, Sigma-Aldrich, USA), phosphoric acid (H_3_PO_4_, 85%, Merck, Germany), zinc chloride (ZnCl_2_, ≥98%, Sigma-Aldrich, USA), and deionized water produced using a GFL Gesellschaft für LabortechnikmbH distiller (Type 2004, Burgwedel, Germany; resistivity 18.2 MΩ cm, Milli-Q system, Millipore, Germany) were employed for solution preparation and pH adjustment. The biomass material employed in this investigation consisted of olive mill solid residues, obtained after the extraction of olive oil from a local olive mill (Algeria, Tizi Ouzou). This waste mainly comprises crushed olive stones and olive peels. Table 1 summarizes the main thermophysical and chemical properties of phenol that are essential for understanding its behavior in aqueous media and its interaction with adsorbent materials. Table 1 summarizes the main thermophysical and chemical properties of phenol that are essential for understanding its behavior in aqueous media and its interaction with adsorbent materials. These parameters provide a fundamental basis for interpreting the adsorption mechanism and predicting the performance of the synthesized activated carbons.^23,24^

**Table 1:** Thermophysical and chemical characteristics of phenol

Property	Approximate value
Common noun	Phenol
Raw formula	C_6_H_6_O
Developed formula	C_6_H_5_–OH
Masse molaire	94.11 g mol^−1^
PKA at 25C°	9.89
Chemical structure	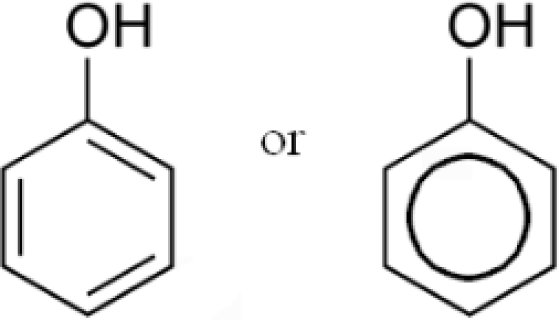
Solubility at 25 °C	93 g L^−1^
Appearance	Colorless to slightly pink crystalline solid
Smell	Sweet, phenolic
Relative density (20 °C)	1.07 g cm^−3^
Melting point	40.5 °C
Boiling point	181.7 °C
Solubility in water (25 °C)	67 (g L^−1^)
Solubility in ethanol, ether, acetone	Highly soluble
Dissociation constant (pKa, 25 °C)	9.95
Henry's constant	3.4 × 10^−7^ atm m^3^ mol^−1^
Log Kow (octanol/water partition coefficient)	1.46

### 2.2. Preparation of activated carbon

The activated carbons were prepared from a lignocellulosic olive pomace precursor using two different chemical activating agents, phosphoric acid (H_3_PO_4_) and zinc chloride (ZnCl_2_), which are the most widely used in the literature,^25,26^ following an identical synthesis procedure. Initially, the raw biomass was thoroughly washed with distilled water to remove adhering dust and surface impurities, then oven-dried at 105 °C for 24 h to ensure complete moisture removal. No grinding or sieving was performed at this stage in order to preserve the natural texture of the precursor. Subsequently, 25 g of the dried biomass was impregnated with each activating agent at a mass ratio of 1 : 1 (activating agent/biomass). The impregnation mixtures were placed in a double-jacketed glass reactor connected to a thermostatic bath, maintained at 100 °C for 6 h to allow for effective chemical activation and interaction between the activating agent and the carbonaceous matrix.^27^ After the activation step, the materials were oven-dried at 110 °C for 24 h, then calcined in a tubular furnace (model Nabertherm) under an inert atmosphere at 500 °C for 1 h, with a heating rate of 5 °C min^−1^, as previously reported by Imad Alouiz *et al.*^18^ The calcined activated carbons were carefully washed several times with distilled water until a neutral pH (6–7) was achieved, ensuring the removal of any residual activating species. The washed materials were then dried again at 105 °C for 24 h, followed by grinding and sieving to obtain a particle size below 200 µm. Finally, the two prepared activated carbons were stored in airtight glass containers at room temperature for further characterization and adsorption experiments. The samples were labelled as OP-AC(PA) and OP-AC(ZC), respectively, according to the activating agent used.

### 2.3. Phenol adsorption experiments

A stock phenol solution was prepared by dissolving 1000 mg of phenol in 1000 mL of double-distilled water, resulting in an initial concentration of 1 g L^−1^. The stock solution was stored in an amber volumetric flask, protected from light, and kept at room temperature to prevent photodegradation. Working solutions of the desired concentrations were obtained by serial dilution of the stock solution using double-distilled water. The pH adjustment of the phenol solutions to the required values was carried out using 0.1 M NaOH and 0.1 M HCl solutions. The pH was accurately measured and controlled using a Hanna Instruments HI2211 pH/ORP meter.

### 2.4. Adsorption study

**Fig. 1:**
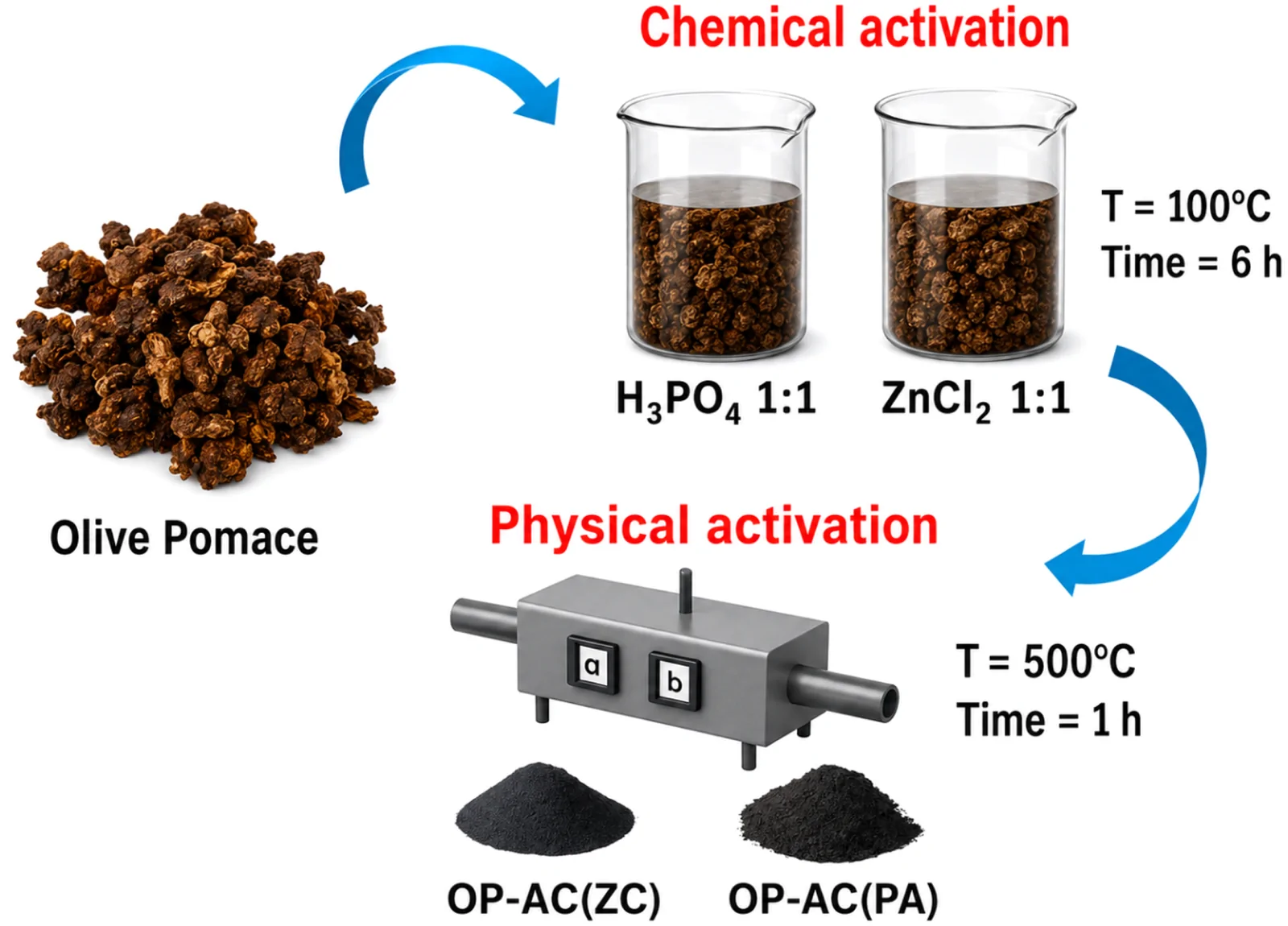
Two-step activation process of olive pomace using H_3_PO_4_ or ZnCl_2_ followed by thermal activation at 500 °C.

The parametric adsorption study of phenol onto the two synthesized activated carbons OP-AC(PA) and OP-AC(ZC), was systematically performed to evaluate the influence of various operational parameters. Initially, the effect of pH was investigated by varying the pH of the phenol solution from 2 to 11, with an initial concentration of 20 mg L^−1^, a solution volume of 50 mL, and an adsorbent mass of 10 mg at ambient temperature for an equilibrium time of 8 h. Subsequently, the effect of adsorbent dosage was examined by varying the mass of activated carbon from 5 to 40 mg, with an increment of 5 mg, using a phenol concentration of 30 mg L^−1^, a solution volume of 50 mL, and the pH maintained at its optimal (free) value as determined in the previous study. The adsorption was carried out at room temperature for 8 h. The effect of initial phenol concentration was then studied using the optimized parameters (free pH), adsorbent mass of 20 mg for OP-AC(PA)and 30 mg for OP-AC(ZC), by varying the initial phenol concentration from 20 to 120 mg L^−1^ in steps of 20 mg L^−1^. For this test, a solution volume of 200 mL was used to allow periodic sampling without altering the system volume. The experiments were performed at ambient temperature for a total contact time of 420 min (7 h), with samples collected at 2, 5, 10, 15, 20, 30, 40, 55, 70, 90, 110, 140, 180, 240, 320, and 420 min to monitor the adsorption kinetics. Finally, the effect of temperature was evaluated under the optimized conditions (free pH), phenol concentration of 30 mg L^−1^, solution volume of 50 mL, and adsorbent mass of 20 mg for OP-AC(PA) and 30 mg for OP-AC(ZC), by varying the temperature from 20 to 60 °C (20, 30, 40, 50, and 60 °C) to assess the thermodynamic behavior of the adsorption process. All adsorption experiments were conducted using high-quality glassware of the SCHOTT DURAN brand. The adsorption tests were performed in Erlenmeyer flasks, and magnetic stirring was provided by ARCO magnetic stirrers. The adsorbent masses were accurately measured using an analytical balance (SHIMADZU AUW220) to ensure the precision of the prepared samples. For temperature-dependent studies, a double-jacketed reactor (LENZ) was used in combination with a thermostatic water bath (LabTech, DAIHAN LABTECH CO., LTD) to ensure precise thermal control throughout the experiments. After reaching adsorption equilibrium, the phenol solutions were centrifuged using an Eppendorf Centrifuge 5804 R to separate the solid adsorbent from the supernatant prior to analysis. The residual phenol concentration in solution was determined using a UV–Visible spectrophotometer (UV-1800, Shimadzu, Japan). The maximum absorbance wavelength (*λ*_max_) of phenol was experimentally determined and fixed at 270 nm. All absorbance measurements were carried out using quartz cuvettes to ensure high optical precision. After recording the absorbance values of the phenol solutions, the corresponding concentrations were calculated using a previously established calibration curve of phenol prepared under identical experimental conditions. The phenol calibration curve was established over the concentration range of 0–120 mg L^−1^, yielding the regression equation: Absorbance = 0.0157*C* (*R*^2^ = 0.99), demonstrating excellent linearity of the analytical method. The removal efficiency (*R*, %) and adsorption capacity (*q*_e_, mg g^−1^) of phenol onto the prepared activated carbons were calculated using the following equations:^28–30^1
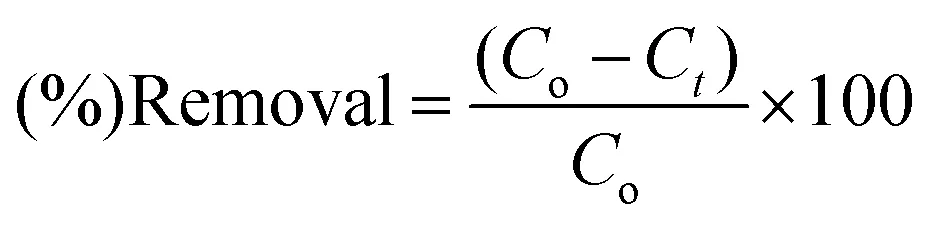
2
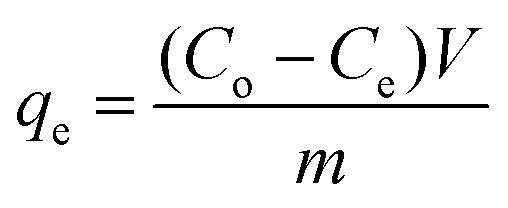
where *C*_0_, *C*_*t*_ and *C*_e_ (mg L^−1^) are the initial, time and equilibrium phenol concentrations, respectively; *V* (L) is the volume of the solution; and *m* (g) is the mass of the adsorbent used. The preparation procedure of the two activated carbons, OP-AC(PA) and OP-AC(ZC), obtained from olive pomace through chemical and physical activation, is presented in Fig. 1.

### 2.5. Characterization studies

To investigate the physicochemical properties of the synthesized activated carbons before and after phenol adsorption, several advanced characterization techniques were employed. The specific surface area, pore volume, and pore size distribution were determined by nitrogen adsorption–desorption isotherms using the Brunauer–Emmett–Teller (BET) method. Measurements were performed with a Micromeritics ASAP 2020 surface area analyzer available at the University of Science and Technology Houari Boumediene (USTHB), Bab Ezzouar. The crystalline structure and phase composition were determined by X-ray diffraction (XRD) using a PANalyticalX’Pert PRO diffractometer operating with Cu Kα radiation (*λ* = 1.5406 Å). The surface morphology and microstructural features were examined by Scanning Electron Microscopy (SEM) using a JEOL JSM-6490LV microscope. Additionally, the surface functional groups and chemical bonding interactions were identified by Fourier Transform Infrared Spectroscopy (ATR) using a PerkinElmer Spectrum Two spectrometer in the range of 4000–400 cm^1^. All these analyses were conducted at the Physico-Chemical Characterization Platform of the University of Science and Technology Houari Boumediene (USTHB), Bab Ezzouar, ensuring high analytical precision and reproducibility of the obtained results.

### 2.6. Determination of the point of zero charge pH_PZC_

The point of zero charge (pHpzc) of the two activated carbons derived from olive pomace OP-AC(PA) and OP-AC(ZC) was determined using the pH drift method.^31^ A series of 0.1 M NaCl solutions (50 mL each) were prepared, and their initial pH values were adjusted to 2, 4, 6, 8, 10, and 12 using 0.1 M HCl or 0.1 M NaOH. Then, 50 mg of each activated carbon was added to the prepared NaCl solutions, and the mixtures were stirred magnetically for 48 h at room temperature. After equilibration, the suspensions were centrifuged to separate the solid and liquid phases, and the final pH of each supernatant was measured. The point of zero charge (pH_PZC_) is identified at the crossing point between the experimental pH_final_*versus* pH_initial_ curve and the 1 : 1 bisector, where the initial and final pH values are equal.

## 3 Results and discussion

### 3.1. Materials analysis

**Fig. 2:**
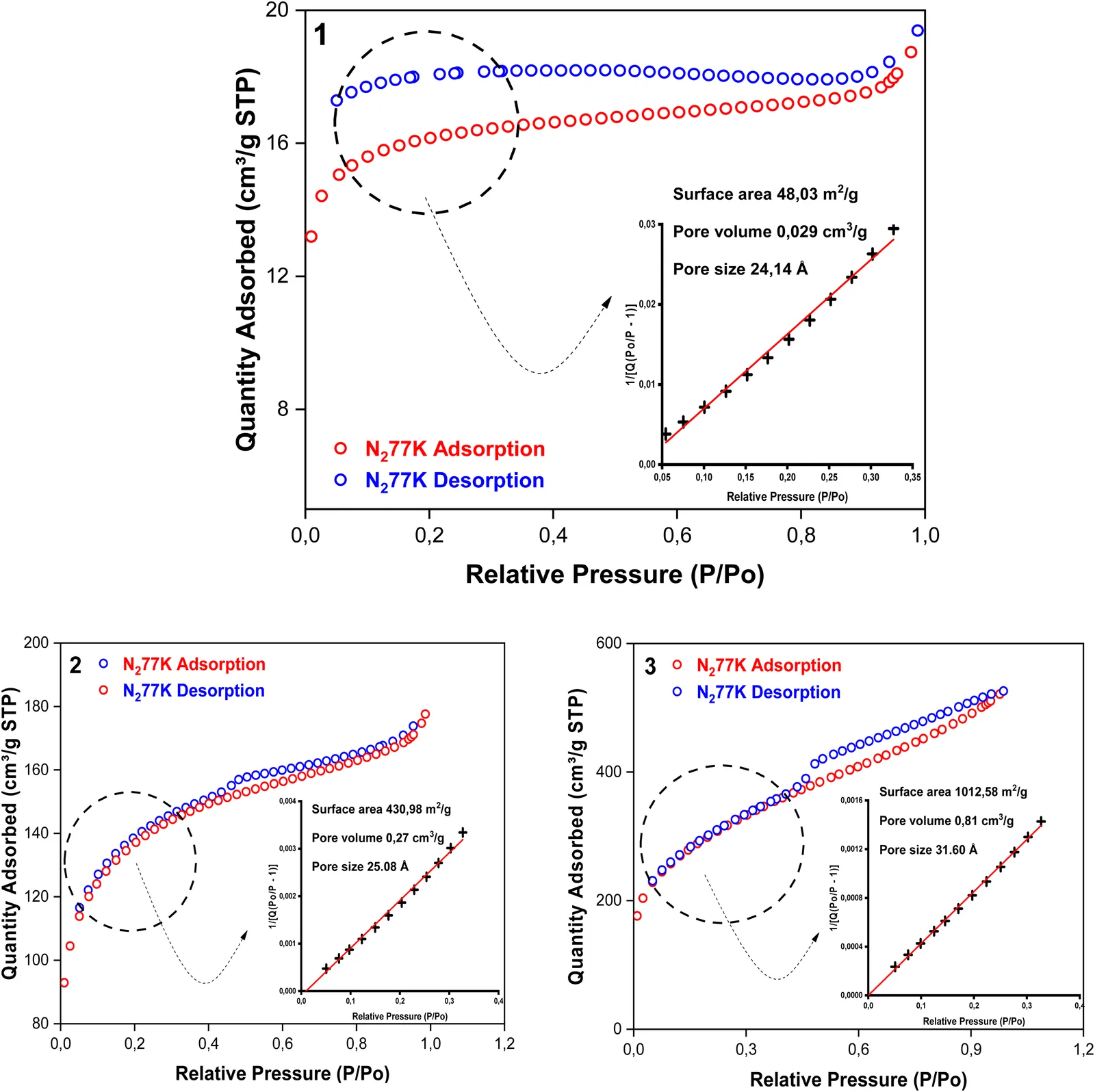
N_2_ adsorption–desorption isotherm at 77 K for Biochar (1), OP-AC(ZC) (2) and OP-AC(PA)(3).

BET the nitrogen adsorption–desorption isotherms of the olive pomace–derived materials (1–3) (Fig. 2) exhibit a clear evolution of the textural structure as a function of the activation treatment. The pristine biochar (1) (Fig. 2) displays a type II isotherm with a negligible hysteresis loop, confirming its essentially non-porous to weakly mesoporous structure, characterized by a very low BET surface area (48.0 m^2^ g^−1^) and limited pore volume (0.029 cm^3^ g^−1^) as reported in Table 1. In contrast, chemical activation drastically enhanced the porosity. The ZnCl_2_-activated carbon (OP–AC(ZC)) (2) (Fig. 2) developed a type I isotherm with a steep adsorption rise at low relative pressure (*P*/*P*_0_ < 0.1), typical of microporous solids, exhibiting a BET surface area of 430.9 m^2^ g^−1^ and a total pore volume of 0.27 cm^3^ g^−1^ (Table 2). This remarkable improvement is attributed to the dehydrating and templating action of ZnCl_2_, which promotes the cleavage of volatile fractions and the formation of an interconnected microporous network. The steep uptake at low *P*/*P*_0_ indicates a predominantly narrow-pore structure, providing accessible adsorption sites for phenol. The average pore size of 25.1 Å may, however, limit phenol diffusion compared with OP-AC(PA). The most outstanding textural properties were achieved for the H_3_PO_4_-activated sample (OP–AC(PA)) (3) (Fig. 2), which presented a type IV isotherm with a pronounced hysteresis loop, revealing a hierarchical micro–mesoporous system. Its BET surface area (1012.6 m^2^ g^−1^), pore volume (0.81 cm^3^ g^−1^), and pore diameter (31.6 Å) (Table 2) highlight the efficiency of phosphoric acid in inducing crosslinking and generating mesopores through phosphate-ester formation and structural swelling during carbonization. This synergistic development of micro- and mesoporosity not only multiplies the accessible surface by more than twenty times compared to the raw biochar but also improves diffusional transport, making OP–AC(PA) a superior adsorbent with exceptional potential for environmental and catalytic applications. The larger pore size (31.6 Å) and pore volume (0.81 cm^3^ g^−1^) of OP-AC(PA) favor greater pore accessibility and phenol diffusion. This is consistent with its higher adsorption capacity (269.03 mg g^−1^) compared with OP-AC(ZC) (143.54 mg g^−1^). The progressive transition from dense carbonaceous residue to highly developed porous frameworks underline the decisive influence of activation chemistry on the morphological evolution of olive pomace–based carbons. Overall, the higher surface area, pore volume, and pore accessibility of OP-AC(PA) provide a more favorable pore architecture for phenol uptake, explaining its superior adsorption performance.

**Table 2:** Textural parameters of olive-pomace derived biochar and activated carbons determined from N_2_ adsorption–desorption isotherms at 77 K

Sample	Isotherm type	*S*, BET (m^2^ g^−1^)	*V*, total (cm^3^ g^−1^)	Average pore size (Å)
Biochar	II	48.0	0.029	24.1
OP–AC(ZC)	I	430.9	0.27	25.1
OP–AC(PA)	IV	1012.6	0.81	31.6

**Fig. 3:**
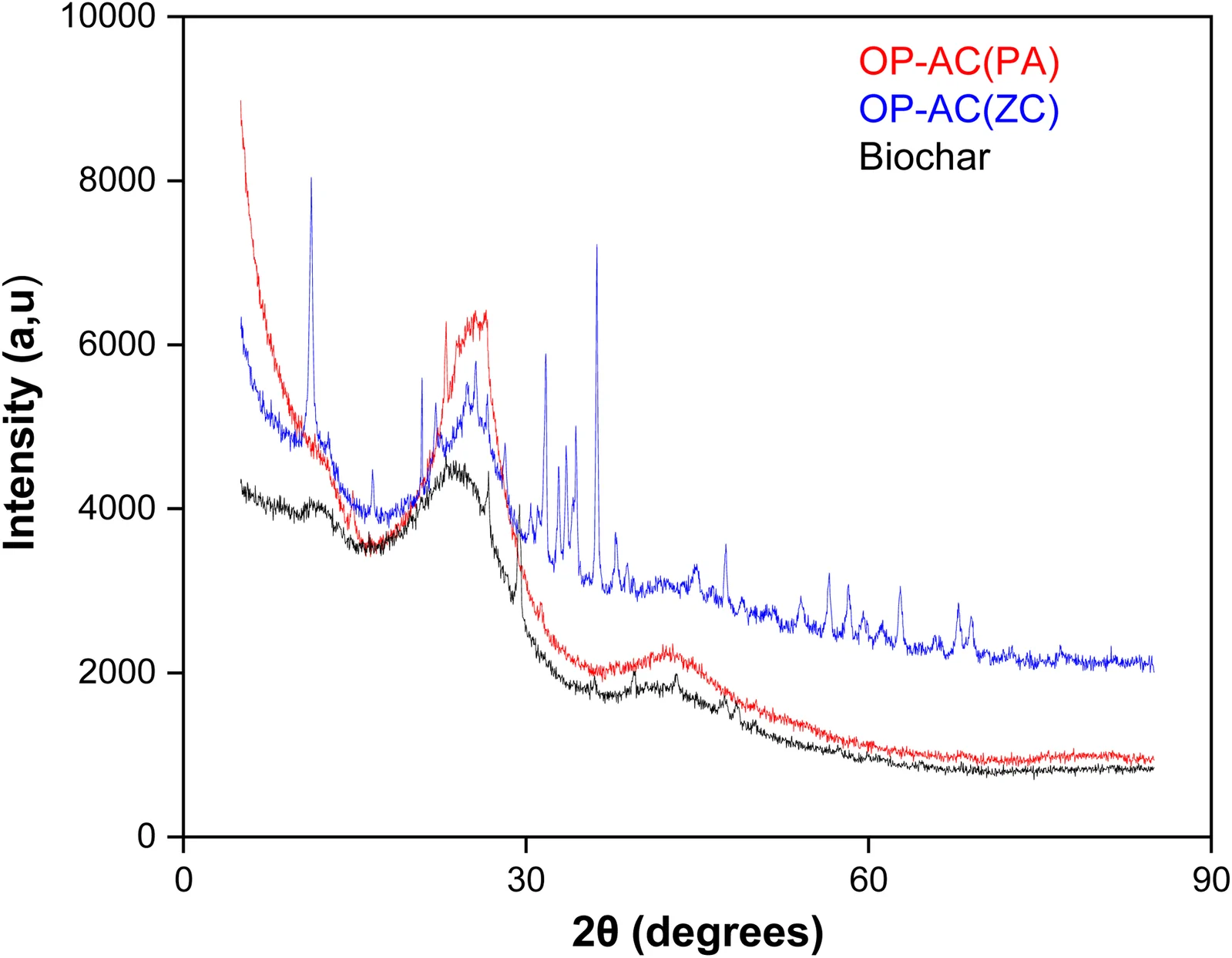
XRD patterns of biochar, OP-AC(ZC) and OP-AC(PA).

The XRD patterns of olive-pomace-derived materials (Fig. 3) show typical amorphous carbon features, characterized by broad (002) and (100) diffraction bands near 24–26° and 43°, respectively. The raw biochar exhibits a broad hump centered around ∼25°, corresponding to an interlayer spacing (*d*002) of approximately 3.56 Å, indicating a poorly ordered, turbostratic structure composed of disordered aromatic layers. This behavior is typical for biomass-derived chars and is consistent with previous studies.^32,33^ Upon ZnCl_2_ activation (OP-AC(ZC)), the (002) reflection becomes slightly sharper and shifts toward a higher angle (from ∼24° to ∼24.7°), yielding a smaller *d*-spacing (∼3.70 Å → 3.60 Å). This shift suggests a partial enhancement of local ordering due to the dehydration and dehydroxylation promoted by ZnCl_2_, which facilitates the rearrangement of carbon layers. However, the presence of distinct diffraction peaks around 31.7°, 34.3°, and 36.1° indicates residual ZnO crystalline phases, a feature commonly reported when Zn species are not fully removed after activation.^34^ In contrast, the H_3_PO_4_-activated carbon (OP-AC(PA)) maintains a broad (002) reflection with no significant shift, confirming that phosphoric acid acts mainly as a cross-linking and pore-forming agent rather than a graphitizing activator. The XRD background is slightly elevated, suggesting the possible formation of amorphous phosphate residues or polyphosphates trapped within the carbon matrix.^35^ This is consistent with the literature describing H_3_PO_4_ activation as a process that promotes microporosity and structural disorder rather than crystallinity.^36^ Overall, the XRD results clearly demonstrate that ZnCl_2_ activation slightly enhances carbon ordering while H_3_PO_4_ activation increases chemical heterogeneity and disorder.

**Fig. 4:**
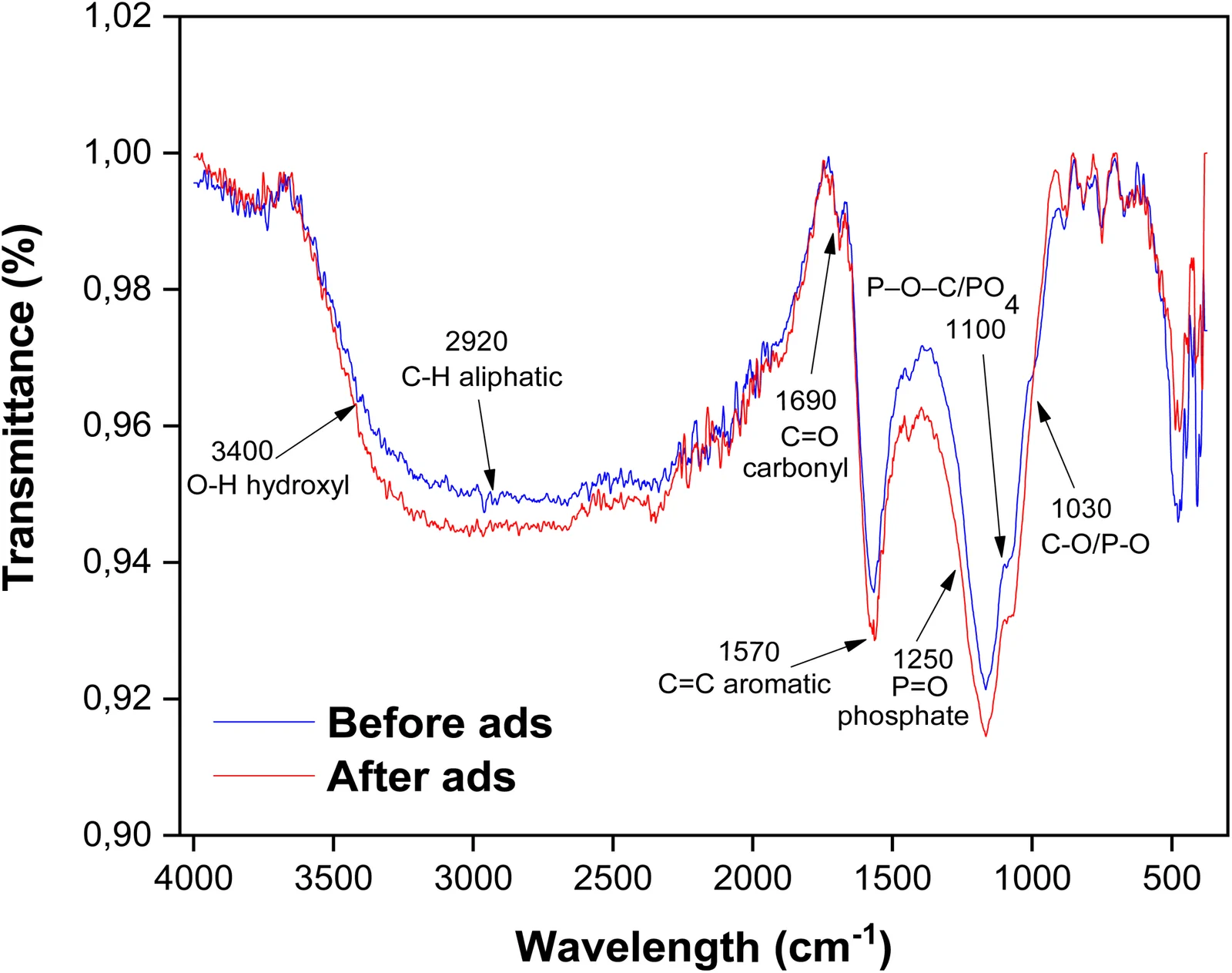
FTIR spectra of H_3_PO_4_-activated olive pomace carbon OP-AC(PA) before and after phenol adsorption.

**Fig. 5:**
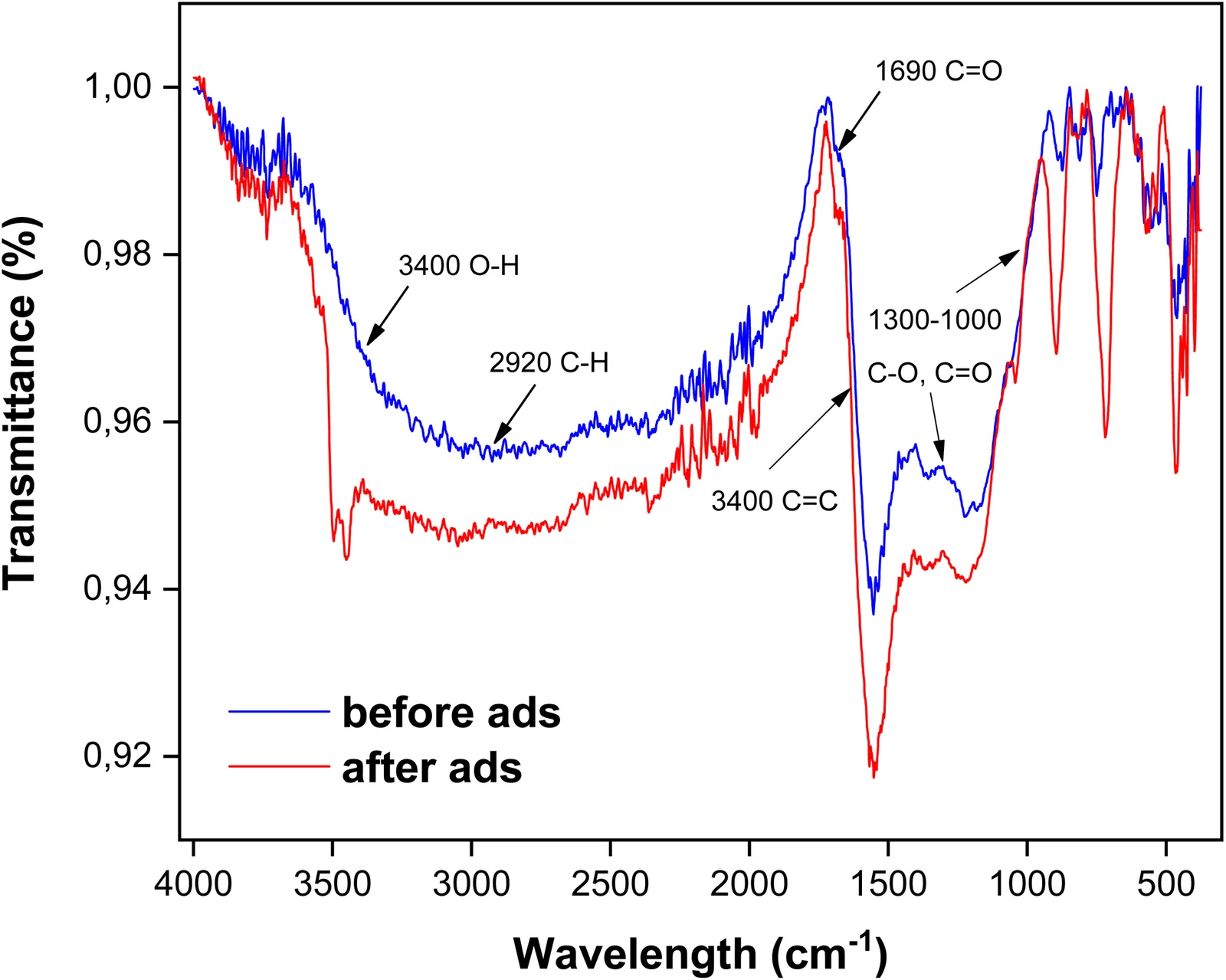
FTIR spectra of ZnCl_2_-activated olive pomace carbon OP-AC(ZC) before and after phenol adsorption.

The FTIR spectra of the H_3_PO_4_-activated olive pomace–based activated carbon (OP-AC(PA)) before and after phenol adsorption revealed clear modifications associated with the involvement of surface functional groups. Before adsorption, the characteristic bands observed at ∼3400 cm^−1^ (νO–H), 2920 cm^−1^ (νC–H), 1690 cm^−1^ (νC=O), and 1600 cm^−1^ (C=C aromatic) indicate the presence of hydroxyl, aliphatic, carbonyl, and aromatic groups, respectively. The strong absorptions in the 1250–900 cm^−1^ region correspond to phosphorus-containing species (νP=O, νP–O–C, and PO_3_ vibrations) originating from phosphoric acid activation.^37–39^ After phenol adsorption, the C=O stretching band shifted from 1691 to 1685 cm^−1^ (Δ = −5.7 cm^−1^), while the aromatic C=C vibration slightly red-shifted (Δ = −2.8 cm^−1^). These changes, together with the intensity decrease of phosphate-related bands (1250–900 cm^−1^), confirm the participation of carbonyl and phosphate oxygen atoms in hydrogen-bond formation with the phenolic hydroxyl groups and the occurrence of π–π interactions between the aromatic ring of phenol and the conjugated domains of the carbon matrix.^40,41^ The overall spectral evolution supports a dual adsorption mechanism, combining specific hydrogen bonding and non-specific π–π stacking, consistent with previous reports for phosphoric-acid activated carbons.^38,42^ The absence of major new bands after adsorption further supports the involvement of existing surface functional groups in phenol uptake. ATR-FTIR analysis of ZnCl_2_-activated carbon before and after phenol adsorption reveals significant modifications in surface chemistry, reflecting strong interactions between the adsorbent and the adsorbate. Before adsorption, the spectrum displays a broad O–H stretching band around 3400 cm^−1^, characteristic of hydroxyl groups and adsorbed water. The weak band near 2920 cm^−1^corresponds to aliphatic C–H stretching, while the distinct absorptions at approximately 1690 cm^−1^ and 1600 cm^−1^ are attributed to C=O (carbonyl/ester) and aromatic C=C vibrations, respectively. Intense bands in the 1300–1000 cm^−1^ range are associated with C–O and C–O–C stretching vibrations of etheric and phenolic groups, which are typically enhanced during ZnCl_2_ activation due to dehydration and cross-linking reactions in lignocellulosic precursors. After phenol adsorption, notable spectral changes are observed. The O–H stretching band decreases in intensity, indicating hydrogen bonding between surface hydroxyls and phenolic groups. The C=O stretching region (∼1690 cm^−1^) exhibits a slight red shift, consistent with hydrogen-bond formation involving carbonyl or ester oxygen atoms and phenolic –OH groups. The aromatic C=C vibration around 1600 cm^−1^ shows minor intensity changes, suggesting π–π stacking interactions between phenol aromatic rings and the conjugated domains of the carbon surface. Furthermore, the attenuation of the 1300–1000 cm^−1^ bands indicates partial coverage or interaction of phenol with surface oxygenated functionalities such as C–O and C–O–C. No distinct Zn–O bands were detected, implying that zinc residues remain either dispersed or encapsulated within the carbon framework and are not FTIR active in this region. Overall, these spectral variations confirm that phenol adsorption on ZnCl_2_-activated carbon proceeds primarily through dual mechanisms: (i) hydrogen bonding between the phenolic hydroxyl and surface oxygenated groups (C=O, –OH, C–O), and (ii) π–π stacking interactions between the aromatic rings of phenol and graphitic domains of the carbon matrix. The results align with previous findings on ZnCl_2_-activated carbons used for aromatic pollutant removal.^43–45^ The limited spectral changes confirm that phenol adsorption occurs without major alteration of the carbon surface chemistry (Fig. 4 and 5).

**Fig. 6:**
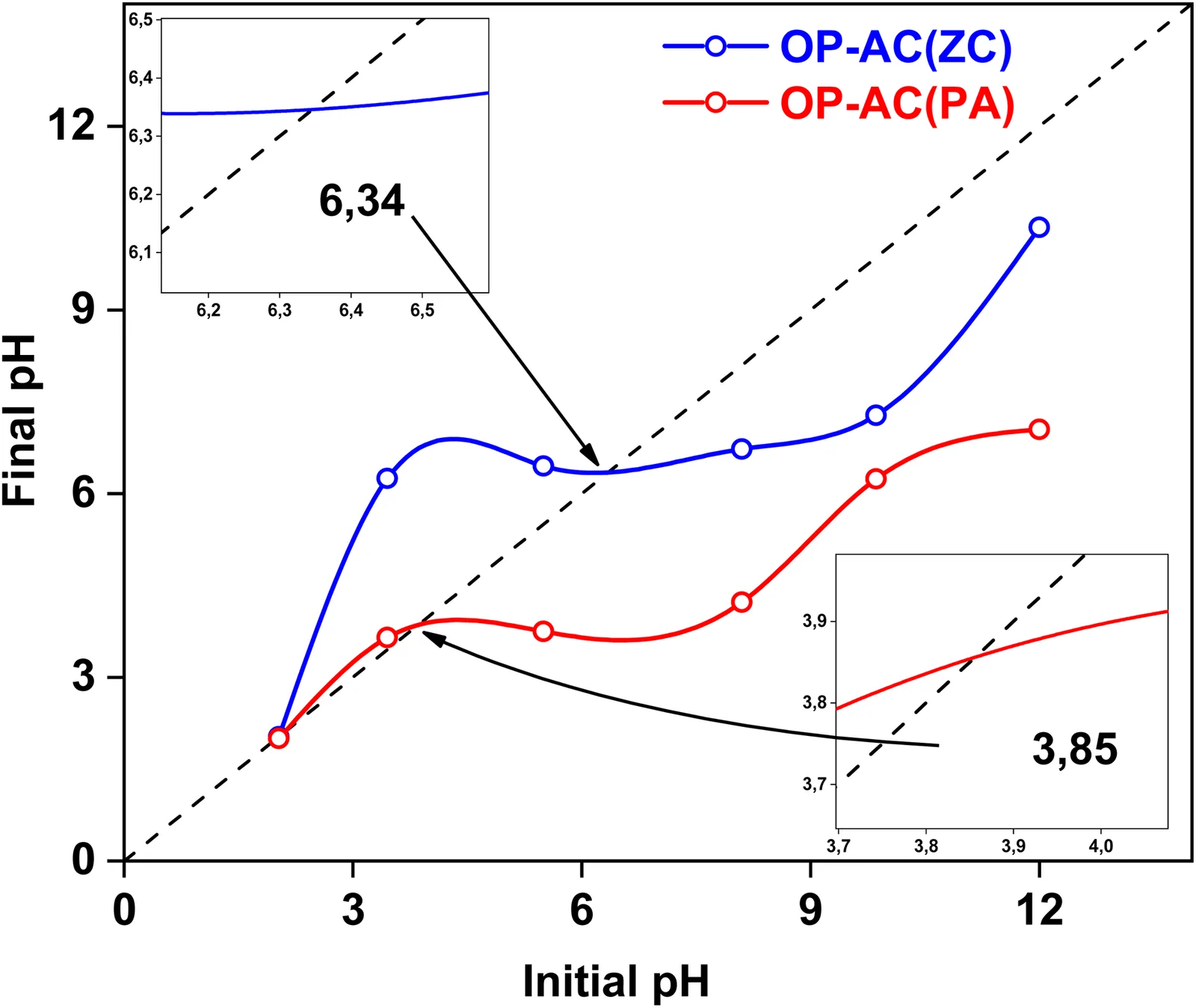
pHpzc determination of OP-AC(ZC) and OP-AC(PA).

Fig. 6 illustrates the pH_PZC_ values, which reveal distinct surface chemistries for the two activated carbons. OP-AC(ZC) exhibited a pH_PZC_ of 6.34, indicating a neutral to slightly basic surface, while OP-AC(PA) showed a lower value (3.85), characteristic of an acidic surface. The higher pH_PZC_ of AC–ZnCl_2_ results from the formation of basic oxygenated groups, whereas the lower pH_PZC_ of AC–H_3_PO_4_ is due to acidic phosphate and carboxylic functionalities. These findings confirm that the activating agent strongly influences the surface charge behavior and adsorption selectivity of the carbons. Below the pH_PZC_, the carbon surface is positively charged, while above the pH_PZC_, it becomes negatively charged.

**Fig. 7:**
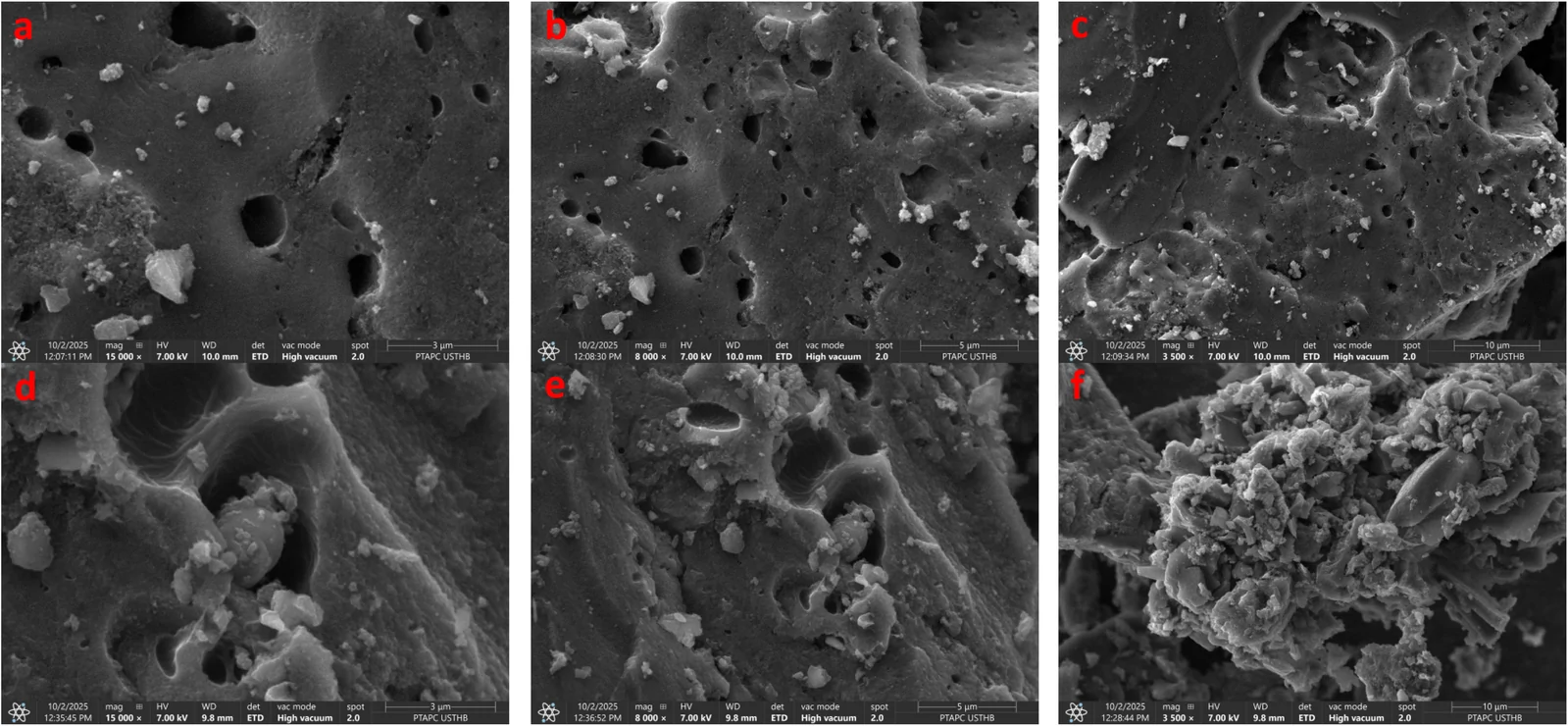
SEM images of OP-AC(PA) before (a–c) and after (d–f) phenol adsorption.

In Fig. 7, we show the surface morphology of OP-AC(PA) before and after phenol adsorption, highlighting the transition from well-defined open pores to partially blocked and smoother pore structures. The OP-AC(PA) surface before adsorption exhibits a highly cavitary morphology with open pore mouths and sharply defined edges, indicating well-developed mesoporosity. After phenol adsorption, the micrographs reveal pronounced wall smoothing, the formation of an organic film, and partial pore-mouth blockage. These changes manifest as reduced surface roughness and textural contrast, consistent with progressive surface coverage. Previously distinct pore structures become less discernible, reflecting partial occlusion. Overall, the morphological evolution confirms effective phenol adsorption and a significant modification of the adsorbent surface. The side-by-side comparison of the SEM micrographs before and after adsorption clearly highlights these morphological changes.

**Fig. 8:**
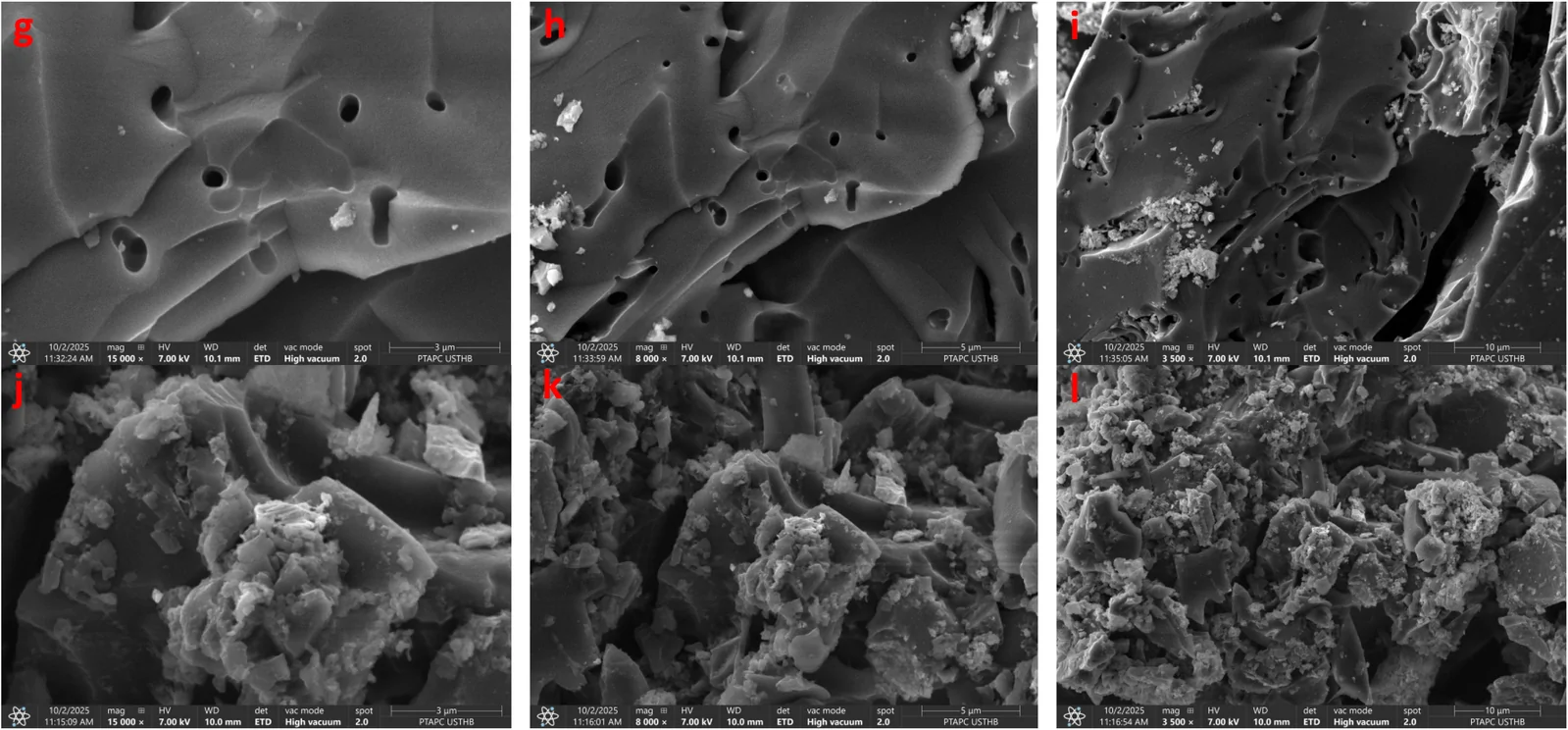
SEM images of OP-AC(ZC) before (g–i) and after (j–l) phenol adsorption.

In Fig. 8, we show the surface morphology of OP-AC(ZC) before and after phenol adsorption, highlighting the transition from well-defined slit-shaped pores to partially filled and smoother lamellar structures. The OP-AC(ZC) displays a lamellar, slit-shaped pore architecture before adsorption, with sharply defined edges and a compact microstructure. After phenol adsorption, the SEM images reveal partial filling of slit-like pores, smoother lamellar surfaces, and thin organic films bridging adjacent layers. These changes result in reduced surface roughness and less distinct pore contours, indicating restricted accessibility of the slit network. The transition from clearly open lamellae to partially occluded structures confirm effective phenol uptake. Overall, the adsorbent undergoes microstructural modifications consistent with pore-mouth blockage and surface coverage by phenolic species. The side-by-side SEM comparison before and after adsorption provides direct visual evidence of these morphological changes.

**Fig. 9:**
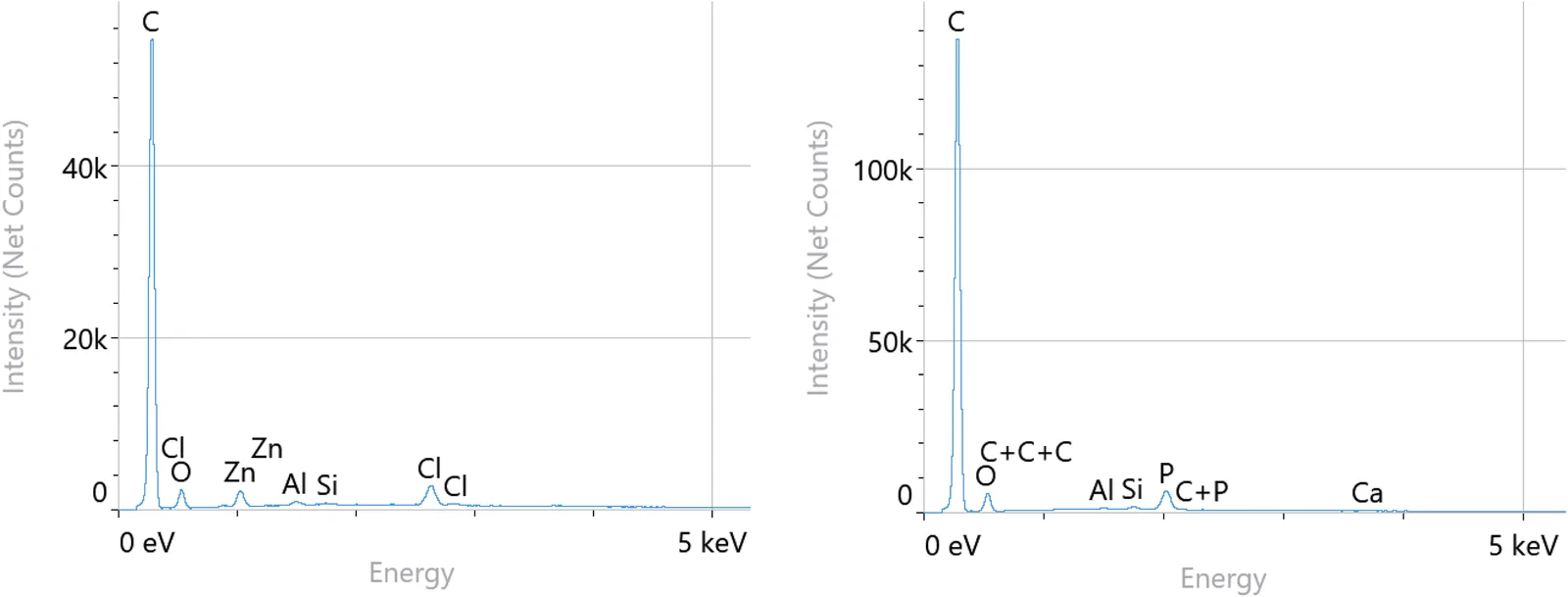
EDS spectra of synthetized OP-AC(ZC) and OP-AC(PA).

The EDS analysis shown in Fig. 9 and summarized in Table 3 reveals that both activated carbons, OP-AC(ZC) and OP-AC(PA), are predominantly carbonaceous (≈80 wt%), confirming efficient carbonization, while the significant oxygen content (11.6–15.9 wt%) indicates the presence of oxygenated surface functionalities. OP-AC(ZC) exhibits measurable amounts of Cl (3.2 wt%) and Zn (4.7 wt%), clearly originating from residual ZnCl_2_and suggesting either partial retention of Zn species or strong interactions with the carbon matrix. In contrast, OP-AC(PA) contains phosphorus (3.0 wt%), evidencing the incorporation of phosphate groups during H_3_PO_4_ activation. Minor levels of Al, Si, and Ca detected in both samples are attributed to inherent mineral ash from the biomass precursor. Overall, the results highlight the decisive effect of the activation reagent on the final elemental composition and surface chemistry of the synthesized activated carbons.

**Table 3:** EDS elemental composition of synthetized OP-AC(ZC) and OP-AC(PA)

Element	OP-AC(ZC)		OP-AC(PA)	
	at.%	wt%	at.%	wt%
C	88.0	79.9	85.9	80.5
O	9.6	11.6	12.7	15.9
Al	0.2	0.4	0.1	0.2
Si	0.1	0.2	0.1	0.3
Cl	1.2	3.2	—	—
Zn	0.9	4.7	—	—
P	—	—	1.2	3.0
Ca	—	—	0.0	0.1

### 3.2. Parametric study

**Fig. 10:**
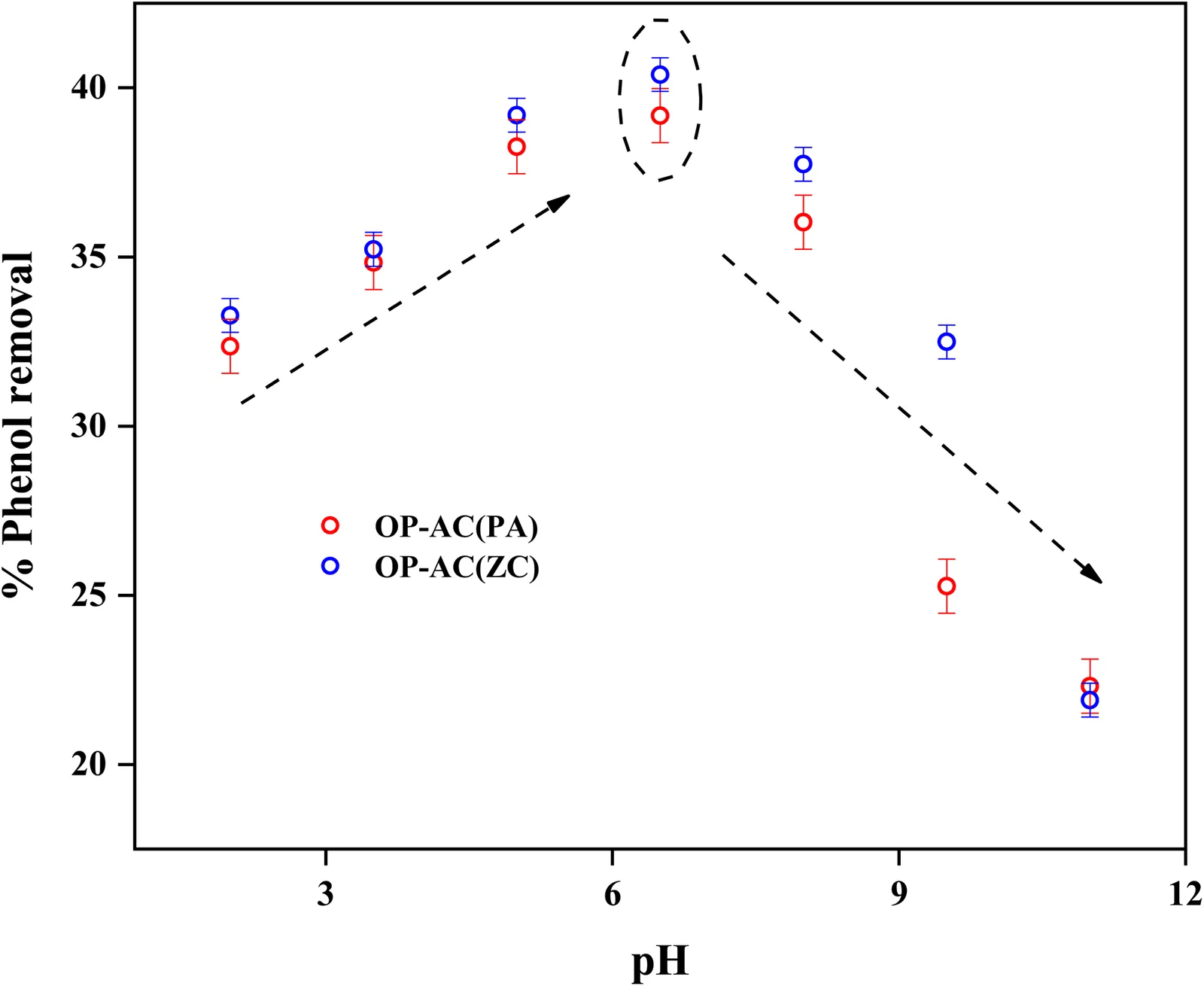
Effect of solution pH on the phenol removal efficiency of OP-AC(ZC) and OP-AC(PA). Experimental conditions: initial phenol concentration = 20 mg L^−1^, adsorbent dosage = 10 mg, solution volume = 50 mL, contact time = 8 h, and ambient temperature.

The effect of solution pH on phenol adsorption was investigated over the pH range of 2–11 (Fig. 10). For both adsorbents, phenol removal progressively increased up to approximately pH 6.5, reaching about 40.5% for OP-AC(ZC) and 39.0% for OP-AC(PA), followed by a gradual decrease at higher pH. This behavior results from the combined effects of phenol speciation, surface charge, and adsorbate–surface interactions. Since phenol (pKa = 9.95) remains predominantly in its neutral molecular form below pH 9.95, the maximum adsorption around pH 6.5 is mainly attributed to favorable non-electrostatic interactions, particularly π–π interactions and hydrogen bonding, together with reduced competition from H^+^ ions. Although the surfaces are negatively charged above their respective pH_PZC_ values (6.34 for OP-AC(ZC) and 3.85 for OP-AC(PA)), significant electrostatic repulsion is not expected at pH 6.5 because phenol is predominantly neutral. At higher pH, particularly near and above the pKa, phenol progressively deprotonates to phenolate, increasing electrostatic repulsion with the negatively charged surfaces and consequently decreasing adsorption. Similar pH-dependent behavior has been reported previously.^46,47^

**Fig. 11:**
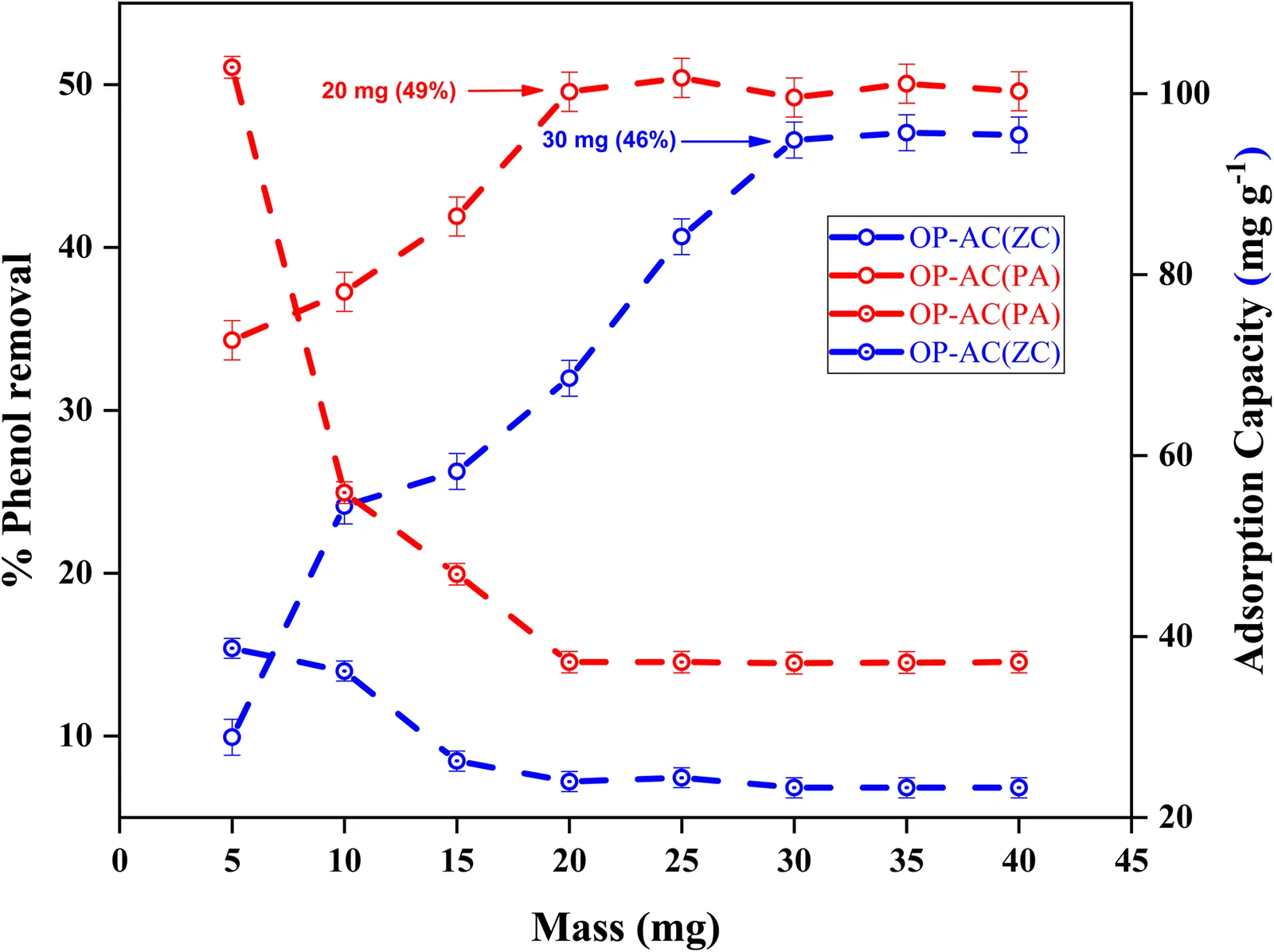
Effect of adsorbent dosage on phenol removal efficiency and adsorption capacity of OP-AC(ZC) and OP-AC(PA). Experimental conditions: initial phenol concentration = 30 mg L^−1^, solution volume = 50 mL, natural solution pH, contact time = 8 h, and ambient temperature.

The results of Fig. 11 show that increasing adsorbent mass enhances phenol removal but decreases adsorption capacity, a trend clearly reflected in the numerical data. For OP-AC(ZC) activated carbon, removal rises from 9.93% at 5 mg to 46.59% at 30 mg, while capacity drops from 38.70 to ∼23.30 mg g^−1^. For OP-AC(PA), removal increases from 34.30% to 49.56% between 5 and 20 mg, with capacity decreasing from 102.91 to 37.17 mg g^−1^. The higher capacities at low masses for H_3_PO_4_/AC indicate a more developed porous structure. Overall, the optimal doses 30 mg for ZnCl_2_ AC and 20 mg for H_3_PO_4_/AC provide maximal removal efficiency.

**Fig. 12:**
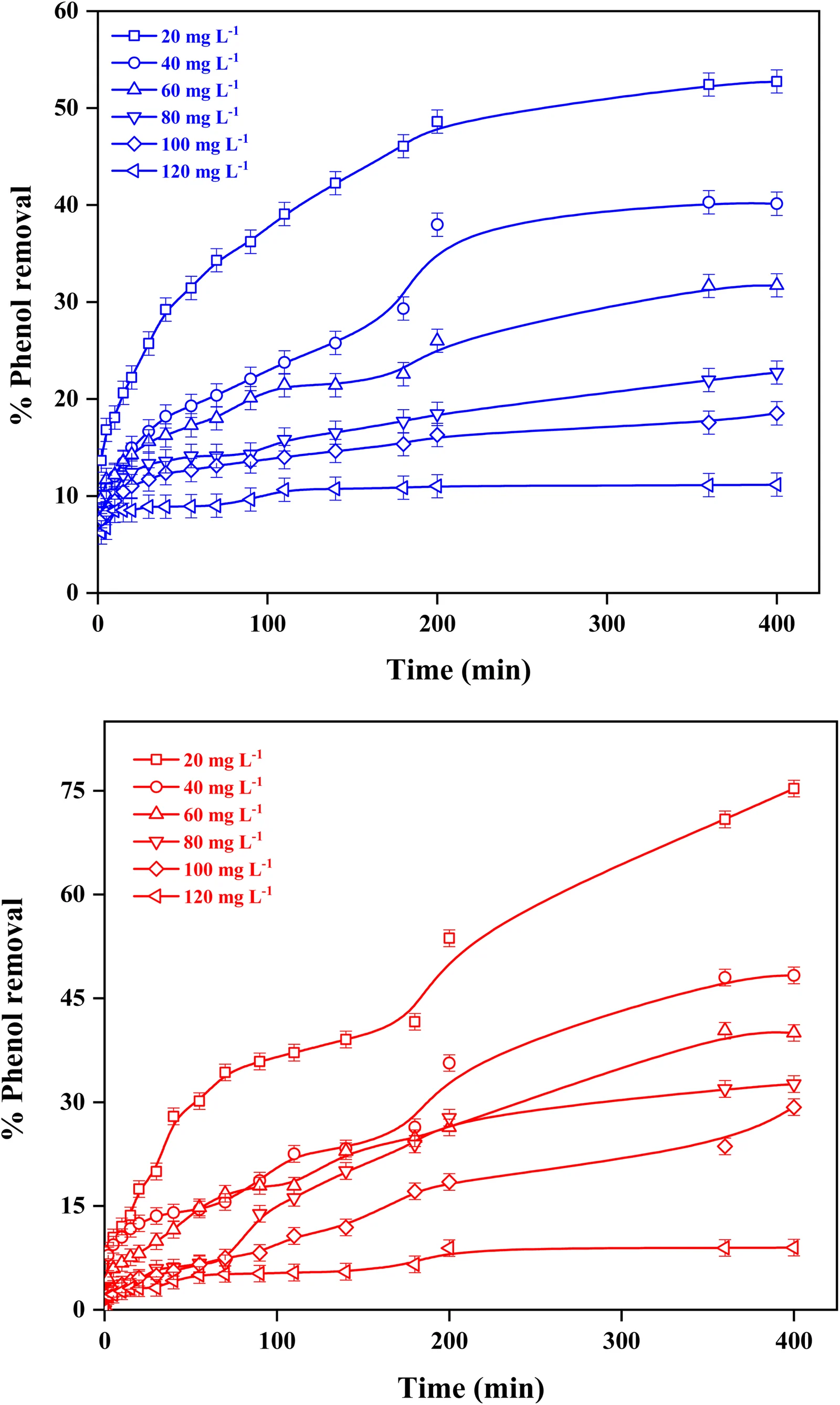
Effect of initial phenol concentration on the removal efficiency of OP-AC(ZC) (blue curves) and OP-AC(PA) (red curves) as a function of contact time. Experimental conditions: initial phenol concentration = 20–120 mg L^−1^, solution volume = 200 mL, natural solution pH, contact time = 7 h, ambient temperature; adsorbent dosage = 30 mg for OP-AC(ZC) and 20 mg for OP-AC(PA).

The effect of initial phenol concentration on its removal by both activated carbons was investigated, and the results are presented in Fig. 12. For OP-AC(ZC), removal increased steadily with contact time but decreased as the initial concentration rose from 20 to 120 mg L^−1^, dropping for example from ≈52.7%to ≈11.2% at 400 min. A similar trend was observed for OP-AC(PA). Where removal decreased from ≈75.85% at 20 mg L^−1^ to ≈8.99% at 120 mg L^−1^ at 400 min. This decline at higher concentrations indicates saturation of active sites and intensified competition among phenol molecules. At low concentrations, both Activated carbons exhibited rapid uptake, reaching over 30% removal before 60 min, reflecting abundant available sites. H_3_PO_4_ activated carbon consistently outperformed ZnCl_2_activated carbon across all concentrations, confirming its more favorable surface chemistry and pore accessibility. Overall, the data demonstrate that increasing initial phenol concentration reduces removal efficiency but enhances the driving force for adsorption kinetics.

### 3.3. Adsorption isotherms

**Fig. 13:**
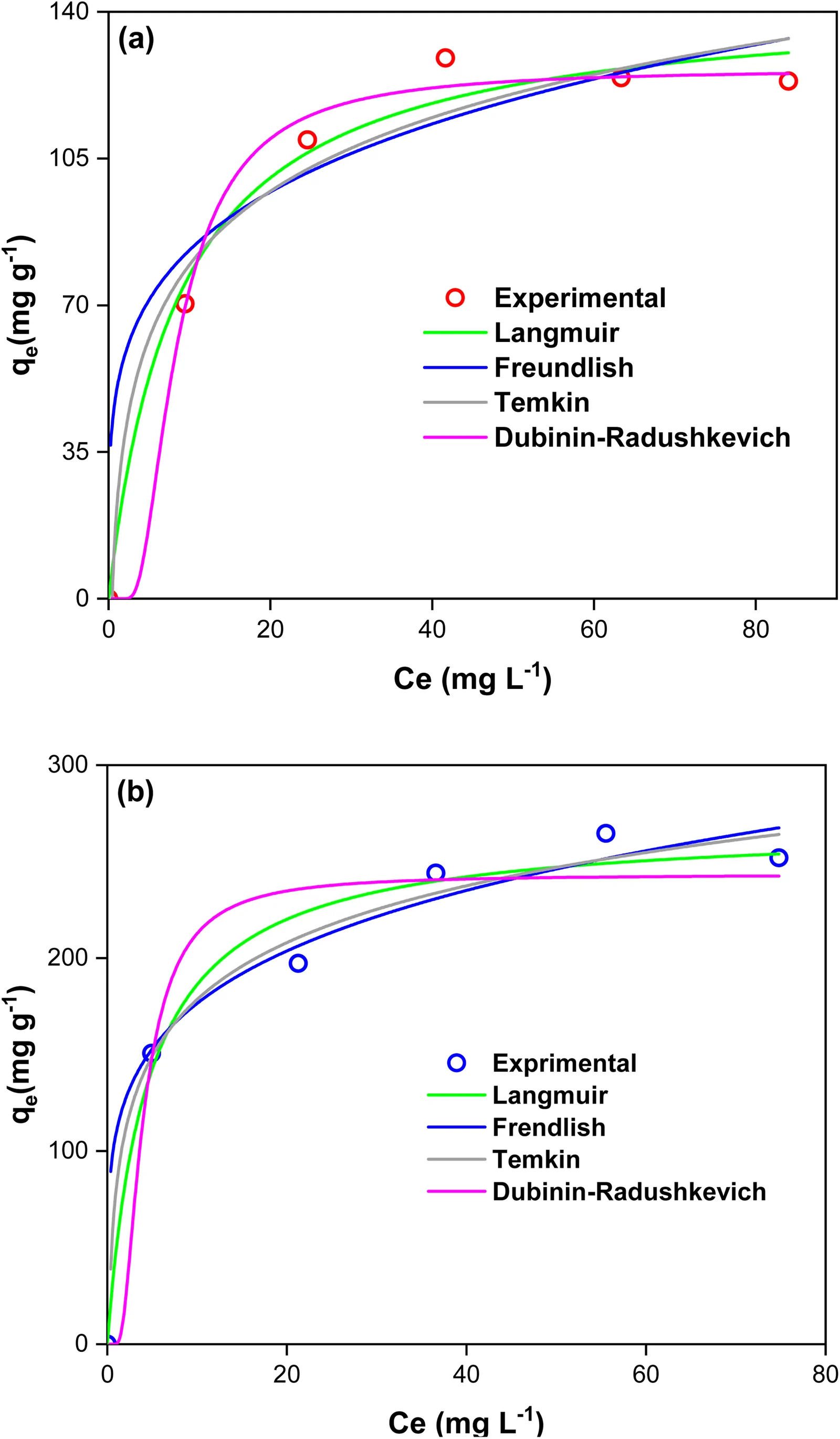
Experimental and modeled adsorption isotherms of phenol onto the two activated carbons OP-AC(ZC) and OP-AC(PA) respectively.

The study of adsorption isotherms is an essential step in understanding the interaction mechanisms between phenol and the activated carbons used as adsorbents. It describes how phenol molecules distribute between the aqueous phase and the solid surface (OP-AC(ZC) and OP-AC(PA)) at equilibrium. To characterize the adsorption behavior of the two activated carbons investigated, several theoretical models were applied, namely Langmuir, Freundlich, Temkin, and Dubinin–Radushkevich (D–R).^48–52^ The parameters obtained from these models, as well as the corresponding equations, are presented in Table 4. These models provide complementary information on the nature of the process (monolayer or multilayer), surface homogeneity, adsorption energy, and the dominant mechanism (physical or chemical).^53,54^ The analysis of the parameters derived from these isotherms makes it possible to evaluate the efficiency of the adsorbents, compare their performances, and better understand the phenol–surface interactions. The adsorption isotherm modeling of phenol onto the two advanced prepared activated carbons OP-AC(PA) and OP-AC(ZC) shown in the Table 4 and Fig. 13 and revealed significant differences in phenol removal performance and surface affinity. The Langmuir model, which assumes monolayer adsorption onto homogeneous sites,^55,56^ demonstrated a maximum adsorption capacity of 269.033 mg^−1^ for the OP-AC(PA), markedly higher than that of the OP-AC(ZC) 143.542 mg g^−1^, indicating a superior adsorption efficiency. The Freundlich model, which reflects adsorption on heterogeneous surfaces,^57,58^ confirmed this trend, with the Freundlich constant being considerably higher for OP-AC(PA) 109.66 compared to ZnCl_2_ 49.78, suggesting a more favorable surface for phenol uptake. Similarly, the Temkin model, which accounts for adsorbent–adsorbate interactions, revealed stronger interactions for OP-AC(PA) *b* = 6.6673 L g^−1^; *B* = 42.501 J mol^−1^ than for OP-AC(ZC) *b* = 2.316 L g^−1^; *B* = 25.34 J mol^−1^, highlighting the higher adsorption energy and stability of the former. The Dubinin–Radushkevich (D–R) model further supported these findings. With maximum adsorption capacities of 243.13 mg g^−1^ for OP-AC(PA) and 126,30 mg g^−1^ for OP-AC(ZC), in agreement with Langmuir predictions. Overall, while both activated carbons exhibited considerable potential for phenol removal, the H_3_PO_4_-activated carbon consistently outperformed ZnCl_2_-activated carbon across all isotherm models, confirming that the nature of the chemical activating agent plays a decisive role in determining pore structure and adsorption efficiency.

**Table 4:** Adsorption isotherm models and corresponding parameters for phenol removal using the two activated carbons OP-AC(PA) and OP-AC(ZC)

Model	Equation	Parameters	OP-AC(PA)	OP-AC(ZC)
Langmuir	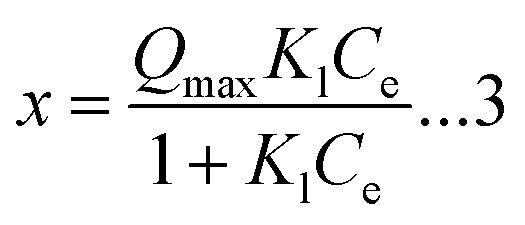	*Q*_max_ (mg g^−1^)	*Q*_max_ = 269.033	*Q*_max_ = 143.542
		*K*_l_ Langmuir constant (L mg^−1^)	*K*_l_ = 0.224	*K*_l_ = 0.116
		*R* ^2^ (correlation coefficient)	*R* ^2^ = 0.980	*R* ^2^ = 0.985
Freundlich	*q*_e_ = *k*_F_*C*^1/*n*^_e_ 4	*k*_F_ (mg g^−1^·(L mg^−1^)^−1/*n*^)	*n* = 0.2066	*n* = 0.2228
		1/*n* (intensity)	*K*_f_ = 109.66	*K*_f_ = 49.778
		*R* ^2^ (correlation coefficient)	*R* ^2^ = 0.924	*R* ^2^ = 0.774
Temkin	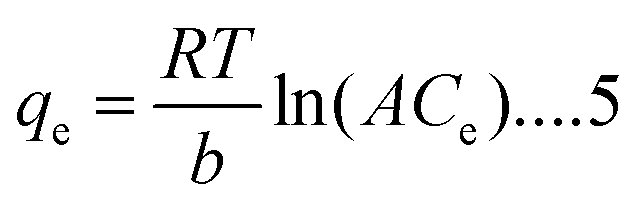	*A* (L mg^−1^)	*A* = 6.673	*A* = 2.316
		*b* (J mol^−1^)	*b* = 42.501	*b* = 25.34
		*R* ^2^ (correlation coefficient)	*R* ^2^ = 0.984	*R* ^2^ = 0.968
Dubinin–Radushkevich (D–R)	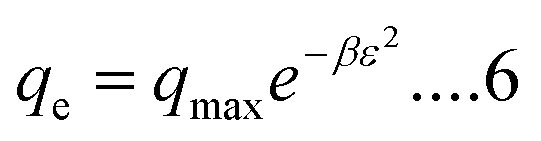	*Q*_max_ (mg g^−1^)	*Q*_max_ = 243.132	*Q*_max_ = 126.305
		Dubinin–Radushkevich constant mol^2^ J^−2^	*β* = 14.738	*β* = 59.076
		*R* ^2^ (correlation coefficient)	*R* ^2^ = 0.957	*R* ^2^ = 0.993

### 3.4. Comparative evaluation of the synthesized activated carbons for phenol removal

To better highlight the performance of the synthesized activated carbons for phenol removal, Table 5 compares the maximum adsorption capacities of the prepared materials with those of recently reported activated carbons derived from various biomass precursors and activated using different chemical agents. The comparison clearly demonstrates the excellent performance of the activated carbons synthesized from olive pomace in the present study for phenol removal. Although OP-AC(ZC) exhibits a moderate specific surface area (430.9 m^2^ g^−1^), its maximum adsorption capacity (143.54 mg g^−1^) remains comparable to or higher than those reported for several ZnCl_2_-activated carbons derived from lignocellulosic biomass. More importantly, the H_3_PO_4_-activated carbon, OP-AC(PA), exhibits a high specific surface area (1012.6 m^2^ g^−1^) and an excellent maximum adsorption capacity (269.03 mg g^−1^), outperforming many recently reported H_3_PO_4_-activated adsorbents for phenol removal, including oakwood (250 mg g^−1^) and *Acacia mangium* bark waste (96.92 mg g^−1^). Although some activated carbons reported in the literature exhibit higher adsorption capacities, they are generally prepared from different precursors and under significantly different activation conditions, making direct comparison difficult. Overall, these results confirm that olive pomace is a highly promising, low-cost, and sustainable precursor for producing efficient activated carbons for the removal of phenol from aqueous solutions.

**Table 5:** Comparison of the maximum adsorption capacities of biomass-derived activated carbons for phenol removal

Materials	Chemical agent	pH and time min	Surface area m^2^ g^−1^	Adsorption capacity mg g^−1^	References
Brazil nu shell	KOH	—	314.3 to 332.2	55.16 and 68.52	59
Oakwood	H_3_PO_4_	4; 120	—	250	60
Cow dung	KOH	6; 180	743.62	518	61
Lignocellulosic agriculture wastes	KOH	4; 10	2490	158.9	62
Bark waste of acacia mangium	H_3_PO_4_	—	390.89	96.92	63
Date palm branches	H_3_PO_4_ and KOH	−; 60	—	77.52	64
Oil-palm shell wastes	KCl and Na_2_CO_3_	—	1433	275.5	65
Rank coal (rawdon)	KOH	−; 220	1951	549.6	66
Tectona grandis sawdust	ZnCl_2_	3.5; 375	585	2.85	67
Vetiver roots	H_3_PO_4_	—	1185	145	68
OP-AC(ZC)	ZnCl_2_	6.5; 400	430.9	143.54	Our study
OP-AC(PA)	H_3_PO_4_	6.5; 400	1012.6	269.033	Our study

### 3.5. Thermodynamic study of phenol adsorption

**Fig. 14:**
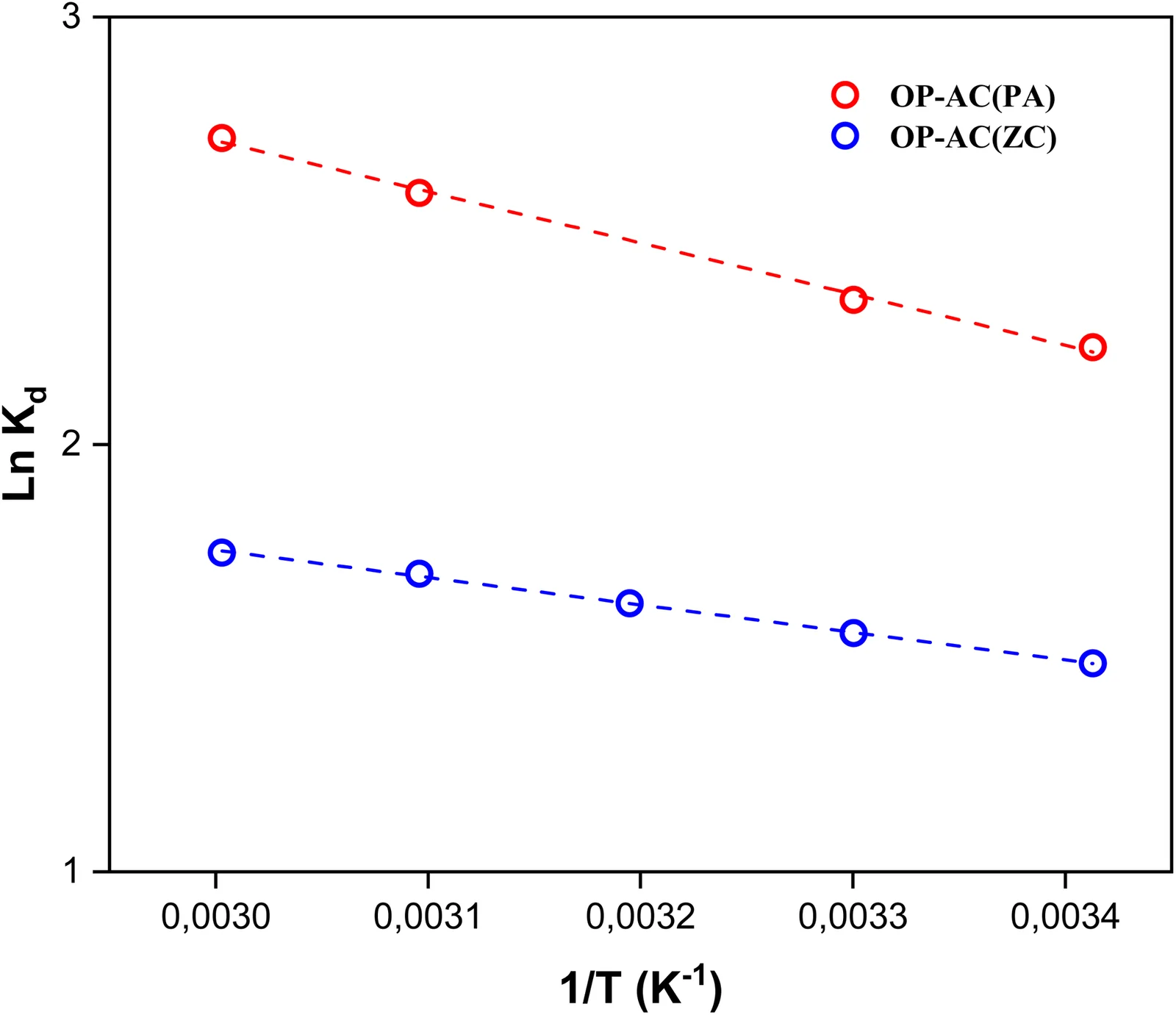
Van't Hoff plots for phenol adsorption onto OP-AC(PA) and OP-AC(ZC).

The thermodynamic parameters of phenol adsorption onto OP-AC(PA) and OP-AC(ZC), including the standard Gibbs free energy change (Δ*G*°), enthalpy change (Δ*H*°), and entropy change (Δ*S*°), were determined to evaluate the energetic characteristics and temperature dependence of the adsorption process (Fig. 14). The standard enthalpy and entropy changes were obtained from the slope and intercept, respectively, of the Van't Hoff plot of ln *K*_d_*versus* 1/*T*, according to eqn (7). Here, *T* is the absolute temperature (K), *R* is the universal gas constant (8.314 J mol^−1^ K^−1^), and *K*_d_ is the adsorption distribution coefficient, calculated as *K*_d_ = *q*_e_/*C*_e_, where *q*_e_ is the equilibrium adsorption capacity (mg g^−1^) and *C*_e_ is the equilibrium phenol concentration (mg L^−1^). The standard Gibbs free energy change was calculated using eqn (9).^69,70^7
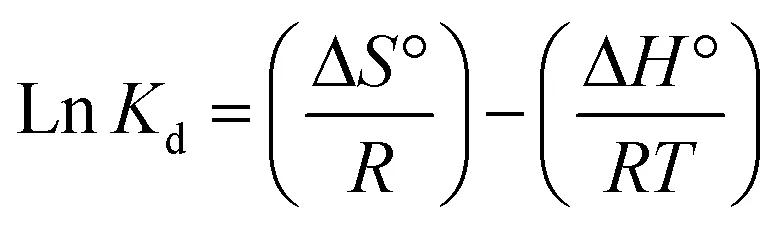
8
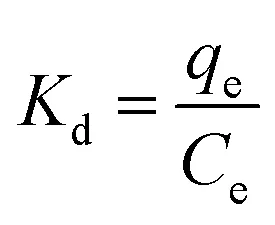

9 Δ*G*° = Δ*H* − *T*Δ*S*°

**Fig. 15:**
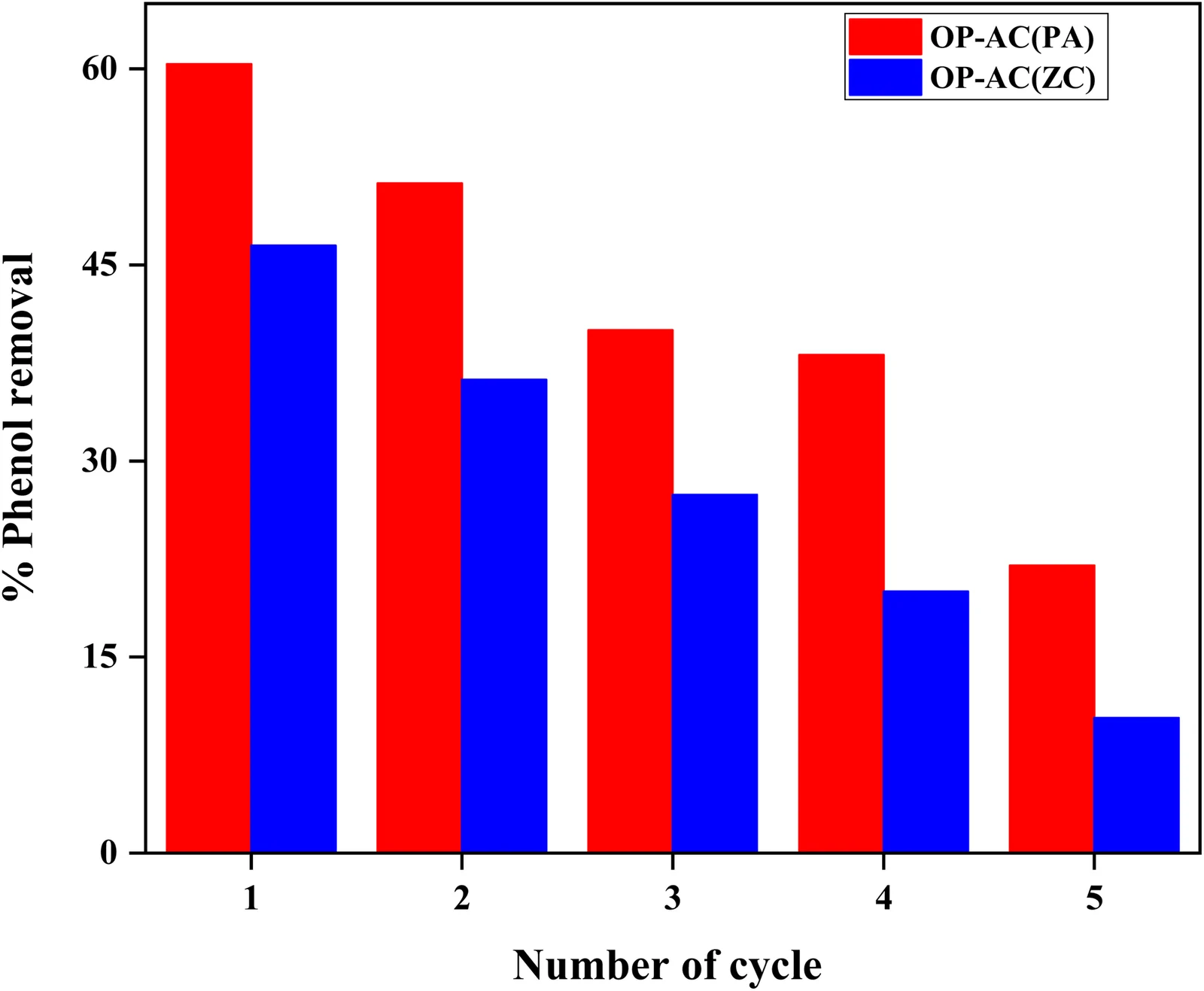
Phenol removal efficiency of regenerated OP-AC(PA) and OP-AC(ZC) over five consecutive adsorption–desorption cycles.

The thermodynamic parameters for phenol adsorption onto OP-AC(PA) and OP-AC(ZC) at different temperatures are summarized in Table 6, while the corresponding variation of Gibbs free energy (Δ*G*°) with temperature is presented in Fig. 15. The negative Δ*G*° values over the investigated temperature range (293–333 K) confirm the spontaneous nature of phenol adsorption. Moreover, the progressive decrease in Δ*G*° with increasing temperature, from −8.445 to −11.445 kJ mol^−1^ for OP-AC(PA) and from −3.450 to −4.650 kJ mol^−1^ for OP-AC(ZC), indicates that adsorption becomes more favorable at higher temperatures. The positive Δ*H*° values of 13.53 and 5.34 kJ mol^−1^ for OP-AC(PA) and OP-AC(ZC), respectively, confirm the endothermic nature of the process, while their relatively low magnitudes are consistent with predominantly physical adsorption. The positive Δ*S*° values (0.075 and 0.030 kJ mol^−1^ K^−1^) indicate increased randomness at the solid–liquid interface, likely associated with the release of water molecules during phenol adsorption. Overall, OP-AC(PA) exhibits more favorable thermodynamic behavior than OP-AC(ZC), consistent with its higher surface area, pore volume, and phenol adsorption capacity.

**Table 6:** Thermodynamic parameters for phenol adsorption onto OP-AC(PA) and OP-AC(ZC) at different temperatures

Material	Temperature (K)	Enthalpy Δ*H*° (kJ mol^−1^)	Entropy changes Δ*S*° (kJ mol^−1^ K^−1^)	Gibbs free energy Δ*G*° (kJ mol^−1^)
OP-AC(PA)	293	13.53	0.075	−8.445
	303			−9.195
	313			−9.945
	323			−10.695
	333			−11.445
OP-AC(ZC)	293	5.34	0.030	−3.45
	303			−3.75
	313			−4.05
	323			−4.35
	333			−4.65

### 3.6. Regeneration and reusability study

The regeneration and reusability of the prepared activated carbons were evaluated over five consecutive adsorption–desorption cycles using ethanol as the desorbing solvent (Fig. 15). Both adsorbents exhibited a gradual decrease in phenol removal efficiency with increasing regeneration cycles. OP-AC(PA) showed better cyclic performance than OP-AC(ZC), with the removal efficiency decreasing from approximately 60% to 22%, whereas OP-AC(ZC) decreased from about 46% to 10% after five cycles. The higher performance retention of OP-AC(PA) indicates better reusability and cyclic stability. The progressive decrease in adsorption efficiency may be attributed to incomplete desorption of phenol molecules and the gradual occupation or blockage of active adsorption sites during repeated cycles. Despite the gradual decrease in performance, both materials retained measurable phenol removal activity after five regeneration cycles, demonstrating their potential for repeated use in phenol removal from aqueous media.

## 4 Conclusion

This study demonstrates that chemical activation plays a decisive role in tailoring the structural, textural, and adsorption properties of olive pomace–derived carbons. ZnCl_2_ activation produces a predominantly microporous material with a BET surface area of 430.9 m^2^ g^−1^ and an adsorption capacity of 143.5 mg g^−1^, while H_3_PO_4_ activation generates a hierarchical micro–mesoporous network with a much higher surface area (1012.6 m^2^ g^−1^) and superior phenol uptake (269.0 mg g^−1^). XRD, FTIR, BET, and SEM–EDS analyses confirm that adsorption performance is governed by the synergy between pore architecture and surface functional groups rather than crystallinity. The presence of oxygenated groups, and phosphorus- or zinc-related surface functionalities, plays a key role in enhancing adsorbate–adsorbent interactions. Phenol removal is driven by combined pore-filling, hydrogen bonding, and π–π interactions, with optimal efficiency observed near neutral pH (pH ≈ 6–6.5). The influence of operational parameters such as adsorbent dosage and initial concentration further confirms the importance of available active sites and diffusion effects in controlling adsorption efficiency. Adsorption isotherms further reveal stronger affinity and higher interaction energy for OP-AC(PA). In comparison with previously reported olive pomace-derived and other biomass-based activated carbons, the prepared materials exhibit competitive to superior adsorption capacities, particularly for the phosphoric acid activated sample. Overall, olive pomace proves to be a highly promising low-cost precursor for producing efficient activated carbons for phenolic wastewater treatment. Future research will focus on the development of dynamic adsorption systems and continuous-flow processes to evaluate the practical performance and long-term stability of the synthesized activated carbons. Furthermore, their application to real wastewater will be investigated to assess their efficiency under complex water matrices and their potential for practical water-treatment applications.

## Author contributions

Djemmali Badreddine: investigation, writing, methodology, formal analysis. Dahdouh Nadjib: supervision, writing – review & editing, interpretation of results, corresponding author. Hafsa Haroun: data curation. Annabel Fernandes: methodology. Venkataraman Sivasankar: validation. Kebir Mohammed: formal analysis. Imene Kezrane: resources. Amina Ouarghi: visualization and Chaabane Toufik: conceptualization.

## Conflicts of interest

The authors declare that they have no conflict of interest.

## Data Availability

The data supporting the findings of this study are available from the corresponding author upon reasonable request.
